# A Retrospective Metabolomics Analysis of Gamma-Hydroxybutyrate in Humans: New Potential Markers and Changes in Metabolism Related to GHB Consumption

**DOI:** 10.3389/fphar.2022.816376

**Published:** 2022-03-03

**Authors:** Tingting Wang, Kirstine L. Nielsen, Kim Frisch, Johan K. Lassen, Camilla B. Nielsen, Charlotte U. Andersen, Palle Villesen, Mette F. Andreasen, Jørgen B. Hasselstrøm, Mogens Johannsen

**Affiliations:** ^1^ Department of Forensic Medicine, Section for Forensic Chemistry, Aarhus University, Aarhus, Denmark; ^2^ Bioinformatics Research Centre, Aarhus University, Aarhus, Denmark

**Keywords:** Gamma-hydroxybutyrate, retrospective study, metabolomics, biomaker discovery, whole blood samples, driving under the influence of drugs (DUID), drug metabolism, UPLC-QTOF analysis

## Abstract

GHB is an endogenous short-chain organic acid presumably also widely applied as a rape and knock out drug in cases of drug-facilitated crimes or sexual assaults (DFSA). Due to the endogenous nature of GHB and its fast metabolism *in vivo*, the detection window of exogenous GHB is however narrow, making it challenging to prove use of GHB in DFSA cases. Alternative markers of GHB intake have recently appeared though none has hitherto been validated for forensic use. UHPLC-HRMS based screening of blood samples for drugs of abuse is routinely performed in several forensic laboratories which leaves an enormous amount of unexploited data. Recently we devised a novel metabolomics approach to use archived data from such routine screenings for elucidating both direct metabolites from exogenous compounds, but potentially also regulation of endogenous metabolism and metabolites. In this paper we used UHPLC-HRMS data acquired over a 6-year period from whole blood analysis of 51 drivers driving under the influence of GHB as well as a matched control group. The data were analyzed using a metabolomics approach applying a range of advanced analytical methods such as OPLS-DA, LASSO, random forest, and Pearson correlation to examine the data in depth and demonstrate the feasibility and potential power of the approach. This was done by initially detecting a range of potential biomarkers of GHB consumption, some that previously have been found in controlled GHB studies, as well as several new potential markers not hitherto known. Furthermore, we investigate the impact of GHB intake on human metabolism. In aggregate, we demonstrate the feasibility to extract meaningful information from archived data here exemplified using GHB cases. Hereby we hope to pave the way for more general use of the principle to elucidate human metabolites of e.g. new legal or illegal drugs as well as for applications in more global and large scale metabolomics studies in the future.

## Introduction

Gamma-hydroxybutyrate (GHB) is an endogenous short-chain organic acid derived from γ-aminobutyric acid (GABA) in the brain and periphery (Struys et al., 2006). GHB is approved as a prescription medication for the treatment of narcolepsy, and in the amelioration of drug and alcohol withdrawal in clinical practice (Carter et al., 2009). Also, GHB, or more recently its lactone prodrug γ-butyrolactone (GBL), is consumed recreationally as a drug of abuse, and is known as a rape drug and knock out drug in cases of drug-facilitated crimes or sexual assaults (DFSA) in forensic toxicology although the frequency apparently is low (Epperson and Ralston, 2016; Francesco et al., 2018). However, the low frequency of detection may be caused by the fast metabolism and thereby narrow detection window of typically up to 6 h in whole blood and 12 h in urine (Busardò and Jones, 2019). Many DFSA cases are likely reported late causing blood or urine samples to be drawn too late for detection of exogenous GHB intake (Kintz et al., 2001; Odujebe et al., 2007; Busardò and Jones, 2019). Therefore, the detection of GHB, discrimination between endogenous and exogenous GHB, and subsequently proving the ingestion of exogenous origin is challenging and likely underreported (Brenneisen et al., 2004; Abanades et al., 2007).

As alternative to direct detection of elevated levels of GHB, reliable and validated biomarkers that reflect prior ingestion of exogenous GHB intake can be useful though such are currently unknown. It is reported that GHB can be metabolized to succinic semialdehyde, followed by oxidation to succinate (Kaufman and Nelson, 1987). GHB can also be further catabolized to acetyl-CoA and glycolate by β-oxidation, and converted to 3-hydroxypropionyl-CoA by α-oxidation (Steuer et al., 2019). In addition, GHB-glucuronide and GHB-sulfate have been reported as Phase II metabolites of GHB (Petersen et al., 2013; Hanisch et al., 2016), but neither are apparently suitable to confirm GHB consumption (Mehling et al., 2017; Piper et al., 2017). Recently, Kraemer et al. (2022), also synthesized fatty acid esters of GHB that also were detected as potentially novel GHB metabolites in blood (Kraemer et al., 2022). GHB-carnitine and GHB-glutamate were tentatively identified for the first time as urinary metabolites of GHB in the study of Steuer et al. (2019), and the structures of GHB-carnitine was later confirmed by an authentic standard in their following study (Steuer et al., 2021). Furthermore, conjugates of GHB with glycine, taurine and pentose were found in urine, and GHB-pentose was reported to be promising for longer detection, while none of these GHB conjugates were found in blood samples (Steuer et al., 2021). 2,4-dihydroxybutyric, 3,4-dihydroxybutyric acid, and glycolic acid have also been reported to be potential GHB biomarkers by a control study with five participants (Jarsiah et al., 2021; Küting et al., 2021). Most of these studies for GHB biomarker discovery were based on a limited number of GHB-users, e.g., to our knowledge up to 20 participants and with a maximum dose of 50 mg/kg reported (Steuer et al., 2021). Furthermore, the time interval from ingestion of GHB to collection of samples is limited with a maximum period of 30 h in a single arm study reporting succinate and glycolate as potential markers based on comparison with pre-intake levels (Palomino-Schätzlein et al., 2017). Novel reliable and importantly validated markers in whole blood is thus still needed for forensic toxicological analyses to confirm exogenous GHB intake.

Untargeted ultra-performance liquid-chromatography-high-resolution mass spectrometry (UHPLC-HRMS) based screening is increasingly used to analyze blood samples for drugs in forensic laboratories (Telving et al., 2016). This technique leaves much unexploited data and in particular if the same quality controlled method has been run over several years, a unique opportunity to mine the existing data for correlations between drug intake and formation of novel metabolites as well as impact on ordinary human metabolism. The feasibility of such a retrospective analysis in metabolomics was initially demonstrated in a seminal paper analyzing data from blood samples from humans exposed to 3,4-methylenedioxymethamphetamine (MDMA) over a 2-year period (Nielsen et al., 2016). The findings provided an initial proof-of-principle that meaningful results can be derived from retrospective data analysis of routine data from toxicological screenings. In contrast to MDMA, GHB is an endogenous compound, and the concept still needs further proof and verification for such more complicated cases. More recently, the principle was also applied by other groups to detect novel direct metabolites of valproate, as well as to examine whether data from post-mortem samples can be used to get insight into mechanism of death (Mollerup et al., 2019; Elmsjö et al., 2020). Still, the method is yet in its infancy and needs further development to, e.g., tackle archived data produced over a longer period, as the shift of retention time (RT) and intensity is much larger in retrospective analysis compared to single or consecutive runs as is custom in the field. Furthermore, a more thorough examination and validation of more advanced data analysis methods is wanted to prepare for future more large-scale studies. Finally, though important insight into direct metabolites of, e.g., MDMA and valproate was demonstrated in previous studies, a validation of the impact of the exogenous compound—here GHB—on endogenous metabolism would ultimately prove that the method merits further attention and use in the future.

Consequently, the aim of this study was to investigate a range of advanced analytical methods to discover those best suited for detecting novel and known biomarkers/direct adducts of GHB consumption in data from routine UHPLC-HRMS screenings. The results examined and potentially validated by comparison to data from controlled studies in the literature. Furthermore, to investigate the impact of GHB intake on human metabolism and also validate this to the literature. For the analysis we used HRMS data from 51 GHB positive and 51 negative driving under the influence of drugs (DUID) blood analysis acquired over a 6 year time period. Towards this aim, data normalization and a range of advanced analytical methods were applied to examine and develop our analytical approach in further depth and demonstrate the power using the GHB data and simultaneously opening up for more large scale metabolomics studies using archived data in the future.

## Experimental Methods

### Chemicals

Acetonitrile (LC-MS grade), methanol (LC-MS grade), formic acid and hydrochloric acid were purchased from Merck (Darmstadt, Germany). Purified water was prepared by a Milli-Q IQ 7000. All other chemical standards including GHB, amphetamine-*d*_5_, cocaine-*d*_3_, diazepam-*d*_5_, and phenobarbital-*d*_5_ were purchased from Sigma-Aldrich (Schnelldorf, Germany). 4-hydroxybutyryl-carnitine chloride was purchased from Toronto Research Chemicals (Toronto, Canada). GHB-glutamate was synthesized following the procedures in Supplementary Material.

### Biological Material

Ante-mortem whole blood samples from drivers suspected of DUID were collected by the Danish police in tubes containing fluoride oxalate mixture and tubes containing a fluoride citrate mixture on different sites in western part of Denmark (4 police districts). The collected samples were subsequently sent to our department by normal mail. All samples were frozen and stored at −18°C immediately after arrival until analysis within a maximum of 7 days.

### Sample Extraction

For the ultra-high-performance-liquid-chromatography quadrupole-time-of-flight mass spectrometry (UHPLC-QTOF) analysis, the extraction procedures followed the method in the study of Telving et al. (2016). An aliquot of the whole blood sample was precipitated with a mixture of methanol and acetonitrile and centrifuged. The supernatant was filtered through a 30 kDa filter and evaporated to dryness, reconstituted and transferred to a LC-vial. For quantitative analysis on ultra-high performance-liquid-chromatography triple-quadrupole (UHPLC-QQQ), the extraction procedures referred to the study of Sørensen and Hasselstrøm, (2012). In short an aliquot of the whole blood sample was precipitated with a mixture of methanol and acetonitrile and centrifuged. The supernatant was transferred to a cation exchange column and the eluate was transferred to a LC-vial.

### Untargeted Screening Using UHPLC-QTOF

The qualitative analysis of the whole blood sample extracts was performed on an ACQUITY I-Class UHPLC system (Waters Corporation, Milford, MA, United States) coupled to a Bruker maXis Impact QTOF mass spectrometer (Bruker Daltonics, Bremen, Germany). The analysis was performed using an ACQUITY BEH C18 (100 mm × 2.1 mm, 1.7 μm) column with mobile phases A consisting of 0.1% formic acid in water and B of acetonitrile, and the analytical method was carried out using the method by Telving et al., (2016).

An electrospray ionization source was operated in positive mode using m/z calibration range of 50–1000 Da at a rate of 10 Hz, and fragmentation analysis was carried out using broadband Collision Induced Dissociation (bbCID) with collision energy of 25 eV. The exactly same analytical method was applied over the 6 years, though the column, was changed regularly e.g. approximately every 6 months during the period. Auto-MS/MS with collisions carried out at energies from 10 to 35 eV was additionally used for some specific fragmentation of selected features after retrospective data analysis for further verification of structures.

### Quantitative Analysis Using UHPLC-QQQ

Quantitative Analysis of GHB was routinely carried out using a validated method with UHPLC-QQQ. The method was described in a previous study (Sørensen and Hasselstrøm, 2012).

### Data Collection and Preprocessing

Samples in this study were collected over 6 years from 2015 to 2020 both inclusive. Fifty-one samples with verified GHB concentrations above 10 mg/kg were matched against a control group of 51 samples with endogenous levels of GHB (<10 mg/kg), 10 mg/kg was selected as a threshold to be sure of GHB intake. Besides GHB, various other drugs were also detected providing both group similar “backgrounds” to level out potential confounding effects by other drugs (Supplementary Table S1). The detailed sample information including the GHB concentration and data collection years of both the control and the GHB positive group is shown in Supplementary Table S2.

Mass spectrometry data obtained from the UHPLC−QTOF were transformed to the *mzml*. file format using Bruker Compass DataAnalysis (Bruker Daltonics, Bremen, Germany) after internal calibration. The *mzml*. files were processed with XCMS in R (version 4.0). The XCMS parameters were optimized (Supplementary Table S3) and a tabulated data matrix list with aligned RT and m/z values was summarized in .csv format. The ions with null value in the data matrix file were imputed with one third of the lowest value of the given ion in all the samples in order to make log-transformation in the next step. Known adducts or isotopes of GHB, namely [(104.0467 + H+1)^+^, (104.0467 + Na)^+^, (104.0467 + Na+1)^+^, (104.0467-H_2_O)^+^, (104.0467-H_2_O+1)^+^] were also excluded in the statistics to get more reliable results for other GHB markers.

As alternative to ordinary quality controls samples in our retrospective analysis, we initially corrected the peak intensity using the internal standards (IS) with the NOMIS method (normalization using optimal selection of multiple internal standards). The data were log-transformed (log 10) to fit the assumptions of the NOMIS method and then normalized by four IS (“metabolomics” package), as illustrated in the study of Sysi-Aho et al. (2007). NOMIS uses variability information from multiple IS to find the optimal normalization factor for each feature. We validated our data quality and the accuracy of the “NOMIS” correction in retrospective analysis by comparing the GHB intensity (not corrected/corrected by NOMIS) in all samples obtained from UHPLC-QTOF to GHB levels measured by UHPLC-QQQ using a linear regression analysis.

### Statistics and Machine Learning

#### T-Test, Fold Change, and PCA

The normalized data obtained by the NOMIS method were used for the following statistical analysis. Pairwise univariate T-tests were used to test the difference for each feature between groups (control/positive) using a critical value of 0.05 (Wang et al., 2021). To account for multiple testing, *p*-values were further adjusted using the false discovery rate (FDR). Also, the fold change (FC) was used to illustrate the ratio of the integrated peak areas between the control and the positive GHB group. Principle component analysis (PCA) by using “ggplot” R package was applied for multivariate analysis.

#### Pearson Correlation Analysis and Correlation Network

Pearson correlation analysis was used to quantitatively calculate the correlation between each feature’s and GHB’s intensity to find potential GHB metabolites. The correlation coefficients were calculated using the “rcorr” function in the “Hmisc” package in R. On the basis of Pearson correlation, we also constructed a correlation network (CN) using all selected GHB-related metabolites to explore the potential interaction between all these features and make a better understanding of the impact of GHB on metabolism. CN can be interpreted as a system biological data analysis method. Correlations between features were considered significant if FDR-corrected *q*-value < 0.1 and only significant features were displayed in the network. Each edge represents correlation between features, and each node represents one selected feature. Features were plotted with the R package “qgraph.” Only networks containing a minimum of three molecules were plotted.

#### Machine Learning Methods

The machine learning methods used for biomarker discovery in this study were selected based on previously reported methods (Nielsen et al., 2016; Liang et al., 2020; Liebal et al., 2020). Orthogonal partial least squares discriminant analysis (OPLS-DA) was applied for feature selection using SIMCA version 16 (Umetrics, Umeå, Sweden). Pareto scaling was applied to the data for the OPLS-DA model. The parameters Q2 and R2X (R2Y) were used to evaluate the performance of the OPLS-DA model. Q2 indicates the prediction quality of the model, whereas R2 explains how well the model fit the data. The accuracy of the OPLS-DA model was validated with 10-fold cross validation, and the dataset was further randomly divided into a training set and a test set containing 50% of the samples for each. Variable importance parameters (VIPs) from OPLS-DA indicate the importance of each feature that contributes to the separation of the two groups (VIP ≤ 0.5: unimportant; VIP > 1: significantly important according to usual interpretation of VIP) (Sinclair et al., 2021). Least absolute shrinkage and selection operator linear regression (LASSO) from the “glmnet” R package was used to predict metabolites associating to exogenous GHB intake. Random forest regression (RFR) and classification (RFC) from the “randomForest” R package were also applied to select the potential metabolites that associated to GHB. RFR calculates the percentage increase of the mean squared error (%IncMSE), which is used to explain the importance of the features corresponding to GHB. %IncMSE indicates the increase in mean squared error (MSE) of predictions (estimated with out-of-bag-CV) as a result of variables being permuted (values randomly shuffled) (27). We also randomized all the samples and applied OPLS-DA model again as an example to see whether we could still identify any markers.

### Metabolite Identification

We used the guidelines from the Metabolomics Standard Initiative to annotate features (Sumner et al., 2007). Selected features with high importance in correlation-based and statistic-based approach were searched from our in-house database with endogenous metabolites (ca. 400 metabolites) and/or online databases as METLIN (https://metlin.scripps.edu), the human metabolome database (http://www.hmdb.ca), lipid maps (http://www.lipidmaps.org), and KEGG (http://www.genome.jp/kegg/) using MetFrag (http://msbi.ipb-halle.de/MetFrag) *in silico* fragmentation for tentative identification. Structures of the selected features were confirmed by matching the m/z-values, fragment pattern, and RT to database or available authentic standards. Annotated metabolites were marked with identification levels. For features identified to level 1, we compared m/z of precursor, retention time and fragmentation spectra to an authentic standard. For level 2 identification, we compared the m/z of precursor, fragmentation spectra to public database.

## Results

### XCMS & NOMIS Align and Normalize the Data Over 6 Years

The GHB concentration quantified by UHPLC-QQQ in the positive group is in the range from 10 mg/kg to 231 mg/kg whole blood, and the GHB level in 51 negative samples are all below 10 mg/kg. RT deviates with a maximum of 20 s before peak alignment, which indicates the variation between samples. Chromatograms of all studied samples before and after RT correction are shown in Supplementary Figure S1. The peak area variation of the four internal standards in all samples varies up to five times (Supplementary Figure S2). Regarding to the integration accuracy, we compared the integrated peak area of GHB by XCMS to the manually integrated peak area of GHB. As shown in Supplementary Figures S3, in general the peak integration accuracy is acceptable (*R* ^2^ = 0.85), and only six samples (11.8%) are a bit off from others with their xcms-integrated peak area around half of the true value.

**FIGURE 1:**
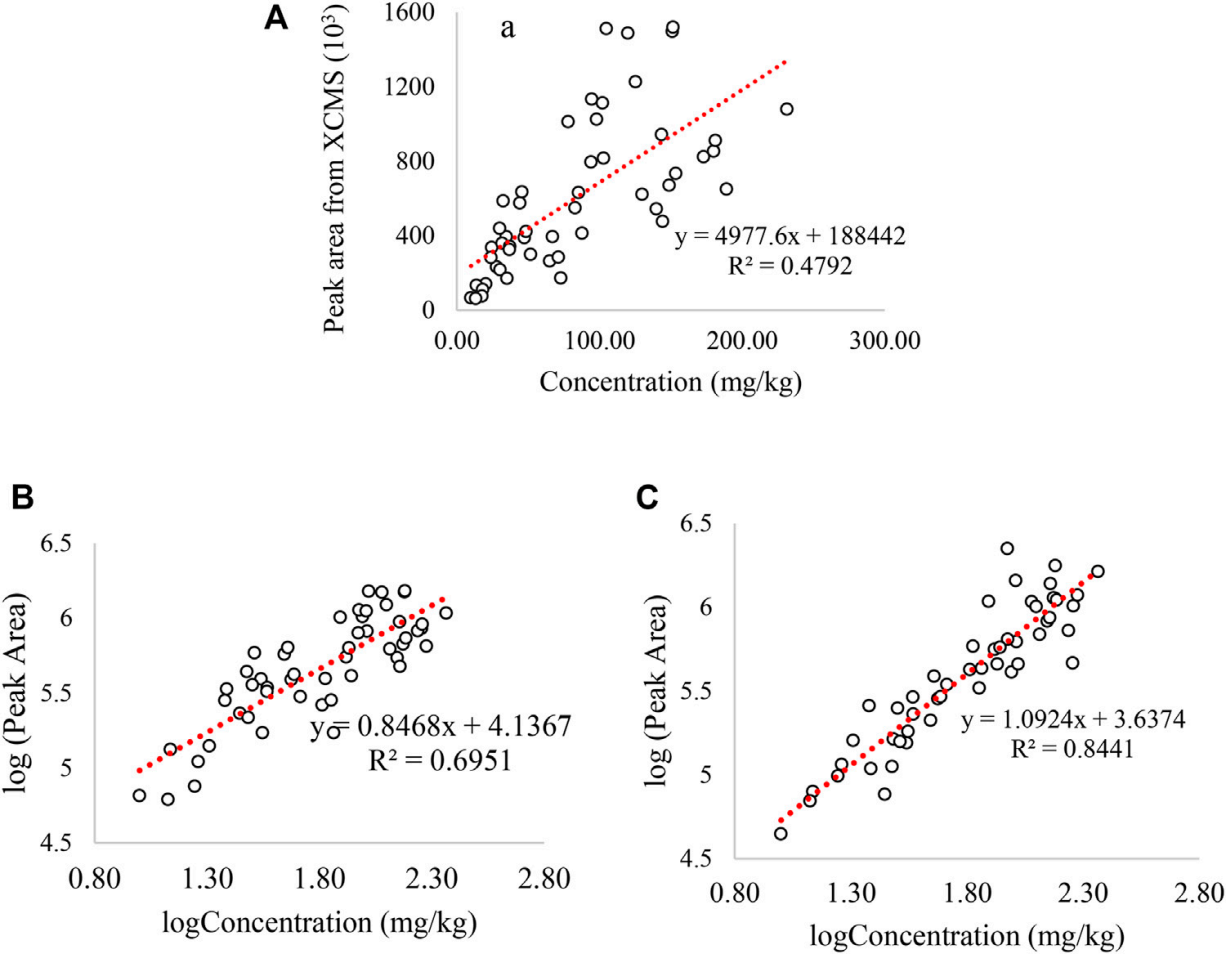
Correlation between the GHB concentration and the GHB peak area integrated by XCMS: **(A)** correlation between GHB concentration and GHB peak area without log-transformation and data normalization. **(B)** correlation between GHB concentration and GHB peak area with log-transformation but without any data normalization, **(C)** correlation between GHB concentration and GHB peak area with log-transformation and NOMIS normalization. The peak area in *y*-axis is from screening method on UHPLC-QTOF, and concentration of *x*-axis is measured by UHPLC-QQQ. Only GHB positive samples were used in all the plots.

The accuracy and performance of the “NOMIS” normalization method in retrospective analysis is evaluated by plotting the logarithmic peak area of GHB in all the positive samples integrated by XCMS using the routine screening data acquired on UHPLC-QTOF to the GHB concentration obtained by UHPLC-QQQ. As shown in Figure 1, the *R* ^2^ is 0.48 before log-transformation and data normalization, and it is 0.69 after log-transformation but before normalization. The correlation further increases to 0.844 following NOMIS, which indicates a strong positive correlation across a long time period and which we evaluate as sufficient for the current purpose.

### Statistical Analysis for Selection of Potential GHB-Markers

**FIGURE 2:**
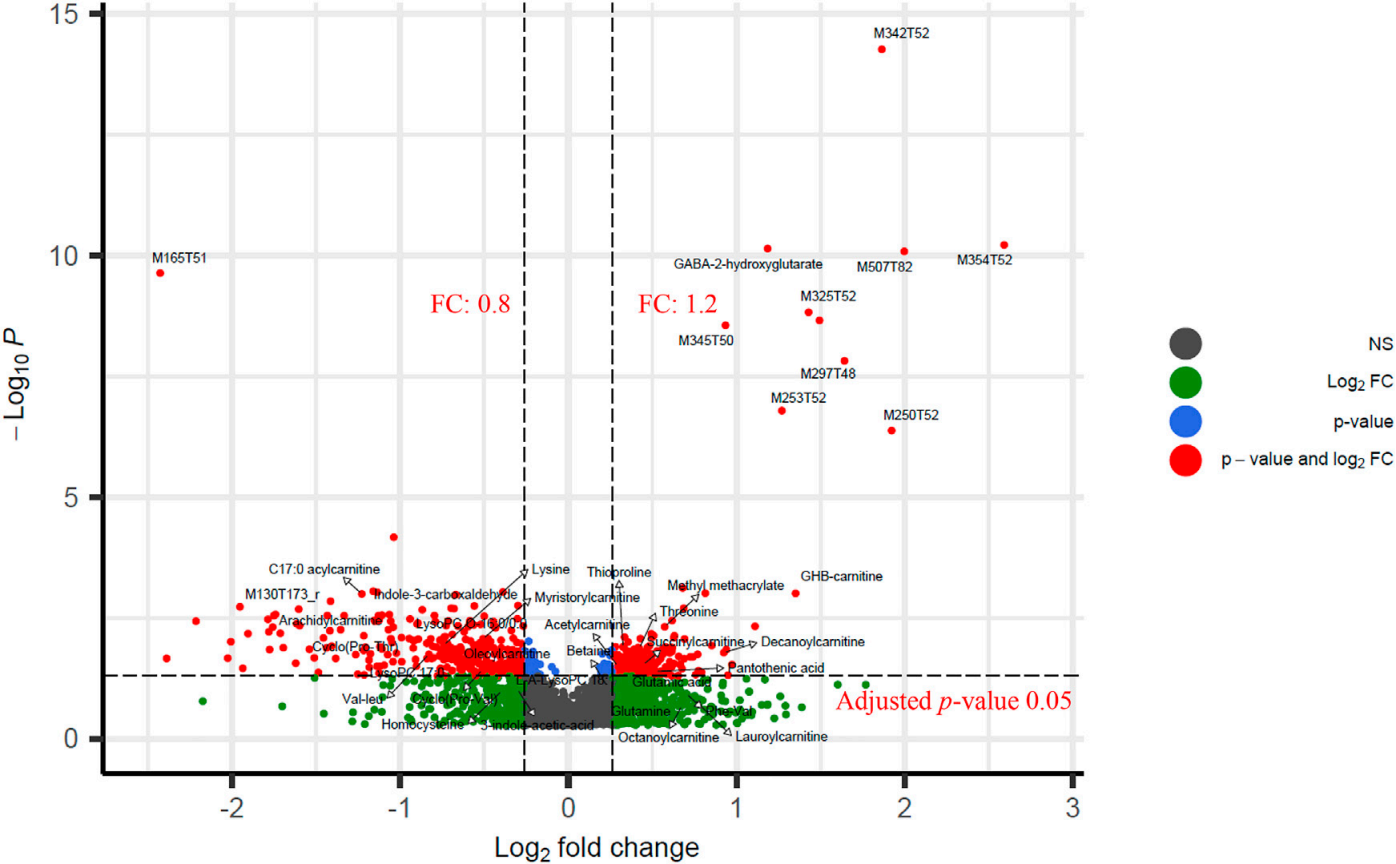
Volcano plot of significant features between the GHB positive group and the control group. *y*-axis represents the log-transformed adjusted *p*-values calculated by *t*-test. *x*-axis is log_2_(FC). Cutoffs of 1.2 and 0.05 are used for fold change and adjusted *p*-values (*q*-value), respectively. FC of 674 features are higher than 1.2, and 516 features have FC lower than 0.8. Adjusted *p*-values of 554 features are higher than 0.05.

XCMS pre-processing extracted 3913 features. The FDR adjusted *p*-values (*q*-value) resulted in 554 features using a threshold of 0.05 and 110 features using a threshold of 0.01. For FC, 674 features were higher than 1.2, 147 were higher than 1.5, and 34 were higher than 2.0. Finally, 516 features had FC values lower than 0.8, and some of them could be potential down-regulated metabolites induced by GHB. M354T52, M507T82, M250T52 are top three features with highest FC, and their FC are 6.0, 4.0, and 3.8, respectively. The FC of all features can be found in Supplementary Table S4. The identified metabolite GHB-carnitine and the tentatively identified metabolite GABA-2-hydroxyglutarate also have relative high FC with values of 2.6 and 2.3 (The top 20 highest). A volcano plot is shown in Figure 2, and FC with 1.2 and *q*-value 0.05 were used as cutoff. PCA shows no clear clustering between the two groups, which indicates that GHB metabolites does not vary enough to affect the first two principal components of the PCA (Supplementary Figure S4).

### Correlation and Machine Learning Models to Select Significant Features Correlated to GHB

**FIGURE 3:**
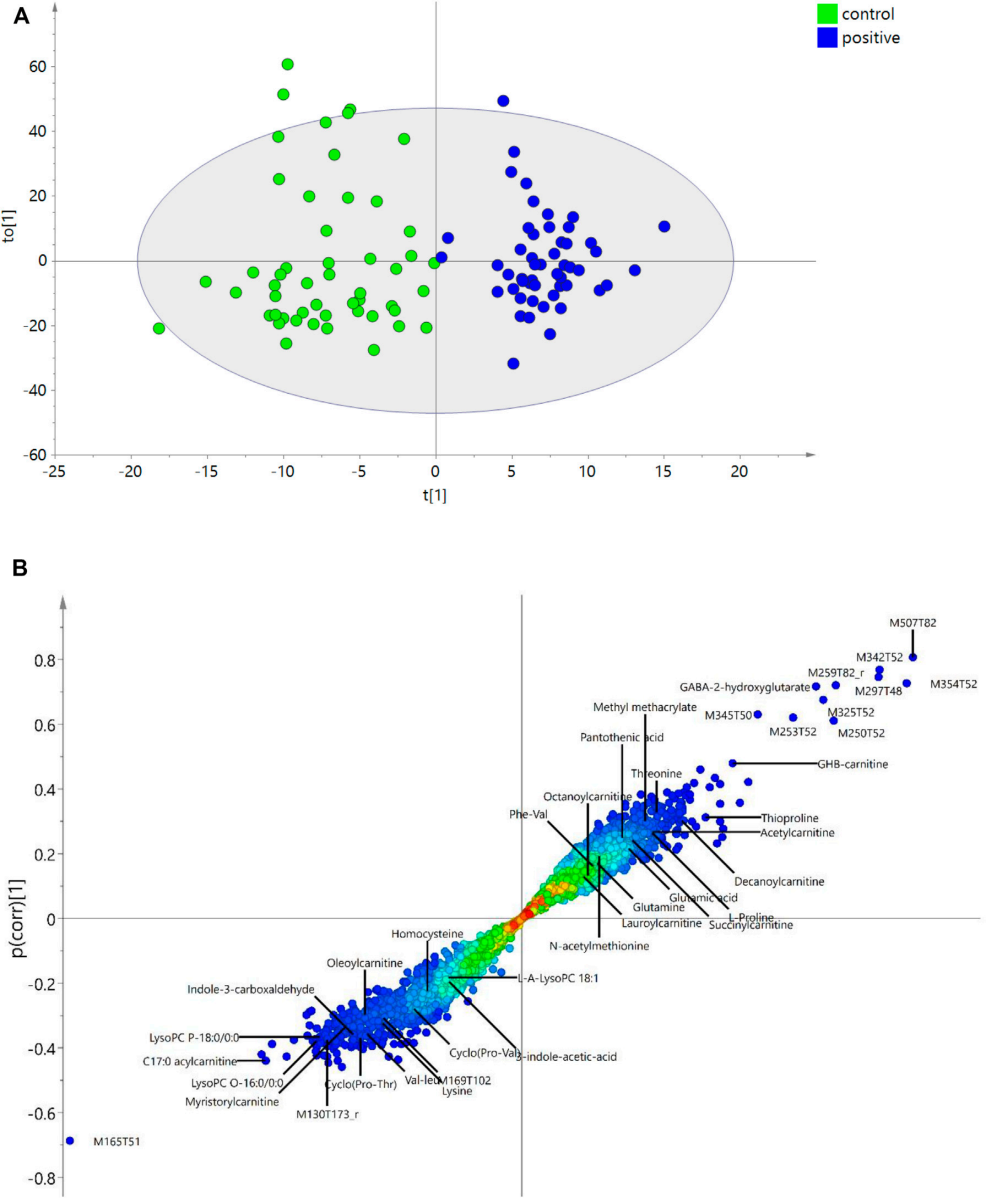
OPLS-DA. **(A)** OPLS-DA plot showing the discrimination between control and the GHB positive group. **(B)** S-plot highlighting the most discriminating features of GHB intake in the positive samples compared to controls in the OPLS-DA model. The *x*-axis shows the magnitude of the variables and their importance, and the *y*-axis indicates the reliability; the closer to ±1, the more reliable. The annotation of each feature with ID refers to Table 1. The unknown features marked with “r” mean they were reported in the literature.

Pearson correlation, OPLS-DA, RFR, RFC, and LASSO were applied to identify the potential metabolites correlated to GHB. In Pearson correlation, features with correlation coefficients (*R*) higher than 0.5 are defined as significant resulting in 11 positively correlated features (*R* > 0.6) and one negatively correlated feature (M165T51) to GHB (*R* < −0.6). These features with high correlation coefficients also show high VIP scores (VIP > 2.5) in OPLS-DA (Figure 3A) and are the most significant features in the S-plot (Figure 3B) and volcano plot (Figure 2).

OPLS-DA discriminates the two groups with R2Y = 0.86, R2X = 0.42, and Q2 = 0.36. The returned root-mean-square error of estimation (RMSEE) is 0.19 using 10-fold cross-validation. The high R2 and the low Q2 indicates some overfitting, which is also seen by the low specificity in the validation set. This could be explained by the endogenous nature of GHB. In OPLS-DA, the sensitivity (true positive) and specificity (true control) from test set validation are 92% and 76%. Among all the features, VIP values of 1350 features are higher than 1.0, and 353 features are higher than 1.5. The importance of features are shown in an S-plot (Figure 3B), where the *x*-axis indicates the magnitude of the variables and their importance, and the *y*-axis shows the reliability, the closer to ±1, the more reliable. The most discriminating features of GHB positive samples versus the control group are highlighted. M165T51, M507T82, M354T52, M342T52, M297T48 are the top five features with highest VIPs that discriminate the two groups. GHB-carnitine, GABA-2-hydroxyglutarate (tentatively identified) and some other endogenous metabolites or reported unknown features also show significant importance in S plot, which will be discussed further in the following sections. As a control of the approach, we finally randomized the united pool of samples and applied the OPLS-DA model again, the results are shown in Supplementary Table S4. As evident, we could not find any GHB markers among the top 50 features with highest VIP score using the randomized samples, e.g., GHB carnitine (M248T57) got a VIP score of only 0.41.

For the LASSO linear regression, the model is first validated by 10-fold cross-validation. The RMSE is 0.27 and the *R* ^2^ is 0.96, which is sufficient to make prediction. LASSO selects 14 features as important features, where four of them (M929T287, M495T277_2, M192T140, M410T173) are found to be insignificant in Pearson correlation with correlation coefficients of 0.36, 0.35, and 0.23, and 0.17, respectively.

The RFR and RFC algorithm is another way to provide information on variable importance. The accuracy of RFC using 10-fold CV is 0.89 (mtry = 58). The RMSE of RFR is 0.62, and *R* ^2^ is 0.80 (mtry = 88). In the results, %IncMSE of 304 features are higher than 1.2, and 76 features are higher than 1.5. The top 11 features are consistent with the top significant features in the Pearson correlation with almost the same order. RFC calculates mean decrease accuracy (MDA) of each feature, which expresses how much accuracy the model losses by excluding each variable. In the results of RFC, MDA of 140 features are higher than 1.2, and 38 features are higher than 1.5. The prioritized features sorted by all the different data analysis methods and how they overlap are shown in Supplementary Table S4. Table 1 shows all the information of selected metabolites that were prioritized and predicted by the different statistics and models. The ID, RT, annotations, T-test, FC, and importance of various models of these features are all shown here, and explanation of the content in this Table 1 will be discussed in the following section.

**TABLE 1:** Metabolites found to be associated with GHB intake that predicted by random forest, Pearson correlation, lasso, and OPLS VIP-Scores.

ID	Annotation	Formula	rt_min	Dir	Idl	FC	m/z	VIP	*p*-value	*q*-value	Lasso	%IncMSE	MDA	PC
M147T21	Glutamine Seo et al. (2018)^a^	C_5_H_10_N_2_O_3_	0.35	↑	1	1.28	147.0764	1.05	0.072	0.126	0	−0.95	−1	0.18
M176T283	3-indole-acetic-acid	C_10_H_9_NO_2_	4.72	↓	1	0.81	176.0706	0.77	0.044	0.095	0	0.54	−1.42	−0.12
M385T74	5-adenosyl- homocysteine	C_14_H_20_N_6_O_5_S	1.23	↑	1	1.15	385.1288	1.29	0.196	0.214	0	−1	0	0.1
M204T23	Acetylcarnitine	C_9_H_17_NO_4_	0.38	↑	1	1.23	204.1238	1.48	0.006	0.031	0	1.39	0	0.26
M456T478	Arachidylcarnitine	C_27_H_53_NO_4_	7.97	↓	2	0.49	456.4044	2.18	<0.001	0.005	0	1	0.31	0.36
M118T24	Betaine	C_5_H_11_NO_2_	0.4	↑	1	1.18	118.0868	1.44	0.036	0.084	0	1.19	1	0.18
M428T460	C17:0 acylcarnitine	C_25_H_49_NO_4_	7.66	↓	2	0.43	428.3737	2.69	<0.001	0.001	0	−0.13	0	−0.38
M199T92	Cyclo (Pro-Thr)	C₉H₁₄N₂O₃	1.53	↓	1	0.52	199.1076	1.63	<0.001	0.008	0	1	1	−0.3
M197T159	Cyclo (Pro-Val)	C₁₀H₁₆N₂O₂	2.64	↓	1	0.7	197.1285	1.09	0.008	0.038	0	1.1	1	−0.23
M316T356	Decanoylcarnitine	C_17_H_33_NO_4_	5.93	↑	1	1.9	316.2493	1.68	0.002	0.017	0	−1	−0.21	0.26
M234T52	GABA-2-hydroxyglutarate	C_9_H_15_NO_6_	0.87	↑	2	2.27	234.0969	2.92	<0.001	<0.001	0.19	4.31	5.57	0.79
M248T57	GHB-carnitine Steuer et al. (2019)^a^	C_11_H_21_NO_5_	0.95	↑	1	2.55	248.149	2.11	<0.001	<0.001	0	2.09	2.37	0.43
M148T21	Glutamic acid Seo et al. (2018)^a^	C_5_H_9_NO_4_	0.35	↑	1	1.3	148.0604	1.29	0.032	0.079	0	−1	−1	0.26
M136T23_2	Homocysteine	C_4_H_9_NO_2_S	0.38	↓	1	0.76	136.0425	1.05	0.051	0.103	0	0.97	−1	−0.2
M146T128	Indole-3-carboxaldehyde	C_9_H_7_NO	2.14	↓	2	0.55	146.0602	1.89	<0.001	0.002	0	−1	−0.37	−0.31
M206T246	3-Indole lactic acid	C_11_H_11_NO_3_	4.11	↓	1	0.85	206.081	0.66	0.042	0.091	0	0.67	1	−0.16
M522T480	L-A-LysoPC; 18:1	C_26_H_52_NO_7_P	8	↓	1	0.77	522.3569	0.88	0.044	0.095	0	−1	0	−0.21
M344T387	Lauroylcarnitine	C_19_H_37_NO_4_	6.45	↑	1	1.68	344.2798	0.77	0.112	0.158	0	−1.08	−1	0.11
M116T22_2	Proline Seo et al. (2018)	C_5_H_9_NO_2_	0.37	↑	1	1.18	116.0711	1.42	0.015	0.051	0	0.83	0	0.26
M120T21	Threonine	C_4_H_9_NO_3_	0.35	↑	1	1.34	120.0656	1.37	0.001	0.014	0	1.46	0	0.32
M147T80	Lysine Seo et al. (2018)	C_6_H_14_N_2_O_2_	1.33	↓	1	0.6	147.1127	1.62	<0.001	0.012	0	1.92	0	−0.27
M510T491	LysoPC 17:0	C_25_H_52_NO_7_P	8.18	↓	1	0.57	510.3559	2.1	0.003	0.021	0	0	1	−0.27
M482T481	LysoPC O-16:0/0:0	C_24_H_52_NO_6_P	8.02	↓	2	0.56	482.3605	2.24	<0.001	0.01	0	−1	−1	−0.32
M508T489	LysoPC P-18:0/0:0	C_26_H_54_NO_6_P	8.15	↓	1	0.57	508.3765	2.23	<0.001	0.012	0	0.37	−1.73	−0.3
M101T88	Methyl methacrylate	C_5_H_8_O_2_	1.46	↑	2	1.49	101.0597	1.27	<0.001	0.005	0	0.9	−0.68	0.45
M298T130	Methylthioadenosine (MTA)	C_11_H_15_N_5_O_3_S	2.17	↑	1	1.89	298.0968	1.57	0.032	0.079	0	−0.34	0	0.19
M372T413	Myristorylcarnitine	C_21_H_41_NO_4_	6.88	↓	1	0.7	372.311	1.97	<0.001	0.008	0	−0.9	0	−0.31
M192T140	N-acetylmethionine Luca et al. (2014)^a^	C_7_H_13_NO_3_S	2.34	↑	1	1.34	192.0689	0.87	0.018	0.058	0.04	1.38	1	0.22
M288T319	Octanoylcarnitine	C_15_H_29_NO_4_	5.32	↑	1	1.6	288.2173	0.95	0.186	0.208	0	−0.84	0	0.09
M426T443	Oleoylcarnitine Luca et al. (2014)	C_25_H_47_NO_4_	7.38	↓	1	0.62	426.3583	1.74	0.002	0.018	0	−0.43	1	−0.26
M220T109	Pantothenic acid	C_9_H_17_NO_5_	1.82	↑	1	1.43	220.1182	1.01	0.01	0.041	0	1.05	−1	0.23
M265T151	Phe-Val	C_14_H_20_N_2_O_3_	2.51	↑	1	1.63	265.1545	0.86	0.064	0.117	0	0.51	−0.64	0.17
M262T41	Succinylcarnitine Steuer et al. (2019)^a^	C_10_H_14_N_4_O_5_	0.68	↑	2	1.36	262.1285	1.29	0.005	0.03	0	−1	0	0.22
M134T23	Thioproline	C_4_H_7_NO_2_S	0.38	↑	1	1.25	134.0271	2.04	<0.001	0.013	0	−1.38	1	0.33
M231T428	Val-leu	C_11_H_22_N_2_O_3_	7.14	↓	2	0.56	231.1743	1.67	0.002	0.018	0	−1.54	0	−0.32
M153T52	Xanthine	C_5_H_4_N_4_O_2_	0.86	↑	1	1.48	153.0407	1.37	0.046	0.097	0	0.98	−1	0.17
M218T81	Unknown		1.34	↓		0.46	218.059	2.76	0.0001	0.004	0	−0.77	0.74	0.37
M80T42	Unknown		0.71	↓		0.14	80.04935	2.63	<0.001	0.011	0	0	1	−0.26
M93T95	Unknown		1.58	↓		0.43	93.06945	2.73	<0.001	0.007	0	1.34	0.96	−0.33
M96T43	Unknown		0.71	↓		0.26	96.0443	2.55	<0.001	0.002	0	−1.02	0	0.32
M79T95	Unknown		1.58	↓		0.22	79.0541	2.5	0.0001	0.004	0	−1	1	0.29
M538T535	Unknown		8.92	↓		0.48	538.3872	2.47	<0.001	0.006	0	1.02	0.62	−0.34
M119T128	Unknown		2.13	↓		0.29	119.0683	2.44	0.0001	0.003	0	−1.08	1.41	0.29
M271T560	Unknown		9.33	↑		1.76	271.2744	2.27	<0.001	<0.001	0	−1.87	0	0.38
M262T52	Unknown		0.87	↑		2.16	262.0134	1.95	<0.001	0.005	0	4.28	2.32	0.54
M256T52	Unknown		0.87	↑		1.53	256.079	1.61	<0.001	0.004	0	1.5	2.84	0.47
M840T313	Unknown		5.22	↓		0.76	840.2059	1.42	<0.001	<0.001	0	1.16	−1.16	0.39
M733T286_3	Unknown		4.76	↑		1.26	733.2255	1.23	<0.001	0.008	0	1.21	0.92	0.4
M130T173	Unknown Steuer et al. (2019)^a^		2.89	↓		0.38	130.0498	2.15	<0.001	<0.001	0	−0.56	0	−0.34
M169T102	Unknown Steuer et al. (2019)^a^		1.71	↓		0.68	169.1331	1.47	0.01	0.042	0	0.03	0	−0.23
M367T466	Unknown Steuer et al. (2019)^a^		7.76	↑		1.11	367.1415	1.05	0.069	0.122	0	0	0	0.15
M259T82	Unknown Steuer et al. (2019)^a^		1.37	↑		2.82	259.0786	3.1	<0.001	<0.001	0	3.91	6.85	0.73
M342T52	Unknown, adduct of GHB		0.87	↑		3.64	342.0608	3.54	<0.001	<0.001	0.17	5.81	7.29	0.83
M165T51	Unknown		0.84	↓		0.19	165.0868	4.48	<0.001	<0.001	−0.13	1.39	4.97	−0.63
M507T82	Unknown		1.36	↑		3.99	507.1547	3.87	<0.001	<0.001	0.33	4.47	6.3	0.8
M297T48	Unknown		0.81	↑		3.12	297.0812	3.54	<0.001	<0.001	0.7	2.76	5.1	0.71
M325T52	Unknown		0.86	↑		2.69	325.1072	2.99	<0.001	<0.001	0.02	5.8	5.12	0.8
M345T50	Unknown		0.84	↑		1.91	345.0675	2.36	<0.001	<0.001	0.02	3.37	4.43	0.65
M354T52	Unknown, adduct of GHB		0.87	↑		6.03	354.0608	3.81	<0.001	<0.001	0.94	6.18	7.11	0.85
M253T52	Unknown		0.87	↑		2.41	253.0213	2.71	<0.001	<0.001	0.13	4.41	3.77	0.75
M250T52	Unknown, adduct of GHB		0.87	↑		3.79	250.0135	3.11	<0.001	<0.001	0	4.33	5.01	0.7

a means the feature has been reported in the literature to be correlated to GHB.

%IncMSE, percentage increase of the mean squared error; MDA, mean decrease accuracy; Dir, direction of regulation by GHB; Idl, identification level. For features identified to level 1, we compared the m/z of precursor, retention time and fragmentation spectra using an authentic standard. For level 2 identification, we compared the m/z of precursor, fragmentation spectra to public database. PC, Pearson correlation coefficients.

### Feature Selection for Further Analysis

Three feature selection strategies were used: a statistic-based approach, a correlation-based approach and a machine learning based approach. In the statistic-based approach, T-test and FC were used, and absolute fold-change (1.2) and *q*-value (0.1) were treated as statistical significance cutoffs. Both correlation and machine learning approaches were based on features with statistical significance. In the correlation-based approach, the top 50 features sorted by Pearson correlation were selected. The top 50 features with highest VIP values in OPLS-DA model were also selected. In addition, all features were matched with our in-house database that includes endogenous metabolites and all the GHB-related metabolites reported in the literatures, and those metabolites showing significance in at least one method (FC > 1.2/FC < 0.8, VIP>1 or *q*-value<0.1) are also listed in Table 1. In the end, all these features with high importance (statistically or the individual top 50 lists) in different strategies and the endogenous metabolites that match with our in-house database and shows significance were combined, resulting in 89 features (Table 1; Supplementary Table S5).

### Metabolites Associated With GHB Levels

A range of features that correlated to GHB levels are identified or tentatively identified, as shown in Table 1, and their fragment patterns that obtained from DDA or DIA mode from UHPLC-QTOF are shown in Supplementary Table S6. In Supplementary Table S6, fragments without abundancy mean that they were fragmented in bbcid mode since not all the precursors got fragmented, otherwise they were fragmented in auto-MS/MS mode. Among the identified features, GHB-carnitine is identified with an authentic standard, which has been reported in previous studies (Steuer et al., 2019, Steuer et al., 2021). GHB-carnitine is found to be a highly significant feature in both OPLS-DA (VIP: 2.11), Pearson correlation (*R* = 0.43), and FC (2.55). M428T460 is tentatively identified as a C17:0 acylcarnitine with VIP score of 2.69 and FC of 0.43, and M456T478 was tentatively identified as arachidylcarnitine. Nine carnitine conjugates are found to be correlated to GHB intake, the levels of myristorylcarnitine, oleoylcarnitine, C17:0 acylcarnitine, and arachidylcarnitine are negatively correlated to GHB according to their FC and Pearson correlation coefficients, while decanoyl carnitine, lauroylcarnitine, octanoylcarnitine, succinylcarnitine, and acetylcarnitine are upregulated by GHB.

Feature M234T52 has the 10th highest VIP among all detected features (2.92) and also high FC (2.27). This metabolite has the same mass as GHB-glutamate that was reported in the study of Steuer et al. (2021), but has so far only been tentatively identified without an authentic standard as it is not commercially available. We therefore synthesized GHB-glutamate according to the structure they proposed in their paper and tested it against our samples (Steuer et al., 2019). However, the RT of the authentic GHB-glutamate did not fit with the RT of M234T52 detected in GHB samples with delta RT of 0.4 min, which means that M234T52 is not GHB-glutamate, but another compound with high correlation to GHB intake, possibly GABA-2-hydroxyglutarate as discussed below. The chromatogram of a GHB sample spiked with authentic GHB-glutamate is shown in Supplementary Figure S5. The two main fragments produced by M234T52 are m/z 84.05 and m/z 130.05, which are also found in GHB-glutamate but with different abundancy (Supplementary Figure S5). Also, GHB-glutamate gives rise to two additional fragments, m/z 102.06 and m/z 69.03, which is not produced by M234T52. The detailed synthesis procedure and NMR spectra of GHB-glutamate are shown in Supplementary Figures S6–S11, and the proposed structure and possible pathway of GABA-2-hydroxyglutarate is shown in Supplementary Figure S12. Two unknown features M259T82 and M507T82 might belong to the same compound due to same RT and show particularly high significance in various statistics and models, but no matches are found in any database.

There are also several amino acids or conjugates of amino acids that are found to be correlated to GHB levels, namely glutamine, lysine, cyclo (Pro-Thr), cyclo (Pro-Val), Phe-Val, 5-adenosyl-homocysteine, homocysteine, threonine, glutamic acid, proline, and Val-Leu. The structures of these amino acids are all confirmed by authentic standards. Lysine, Val-Leu, cyclo (Pro-Thr), cyclo (Pro-Val), and homocysteine are all downregulated by GHB, while the remaining metabolites are upregulated by GHB intake.

### Validation of Different Feature Selection Methods by Comparison to Controlled GHB Studies

**FIGURE 4:**
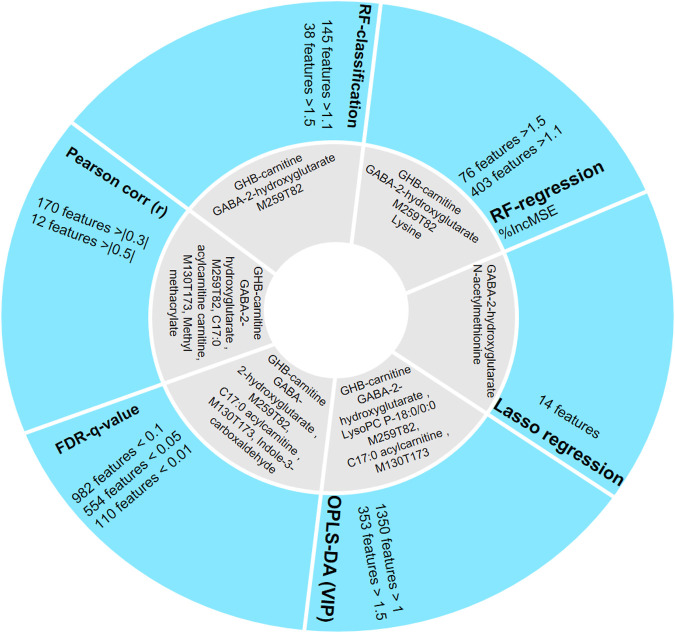
Comparison of the different feature selection methods and identified compounds (as shown in grey area) among the top 50 most important features sorted by each method. FDR *q*-value, OPLS-DA, and Pearson correlation prioritize most metabolites in their top 50 features compared with control studies.

To further compare the performance of the different statistics and machine learning models, the identified GHB-related metabolites among the top 50 features sorted by each method are shown in Figure 4. Some of the identified metabolites have been reported previously in controlled GHB studies and can be used to validate our approach and the different analytical methods.

As GHB-carnitine, succinylcarnitine, and several reported unknowns are not found in LASSO, this method is considered less reliable for biomarker discovery in our case since it penalized these previously described GHB-related metabolites (Liebal et al., 2020). Relatively few GHB-related metabolites are also found in top 50 features sorted by RFR and RFC (Figure 4), with only two GHB-related features (GHB-carnitine and M259T82) consistent with the literature. Both RFR and RFC however prioritize M234T52 (possibly GABA-2-hydroxyglutarate). OPLS-DA is able to model the difference between the GHB positive and control group, and using this machine learning method four reported GHB-related metabolites could be found in top 50 features with high significance (VIP > 1). Pearson correlation provides valuable information to discover metabolites that are highly correlated to GHB intake, and is known as the best method of measuring the association between variables of interest. However, as our study is uncontrolled, Pearson correlation may not be the best method to interpret the results as the Pearson correlation coefficients we obtained from most relevant metabolites are not high enough to take as significant features according to a typical significance cutoff such as 0.5, but still provides additional information for the change of metabolism. FDR *q*-value gives valuable information for potential biomarkers and four of the top 50 prioritized features have previously been identified as GHB-related metabolites (Steuer et al., 2019). Random forest allows direct biological understanding of the decision and classification (Liebal et al., 2020). In aggregate, for our data, the outputs of RFR and RFC vary much compared to the OPLS-DA and FDR *q*-value, which provides less conservative results. Thus, we regard OPLS-DA as the machine learning method used for feature selection giving the most comprehensive information, while its combined use with other statistics such as T-test and Pearson correlation further strengthens the prioritization of relevant features/metabolites.

**FIGURE 5:**
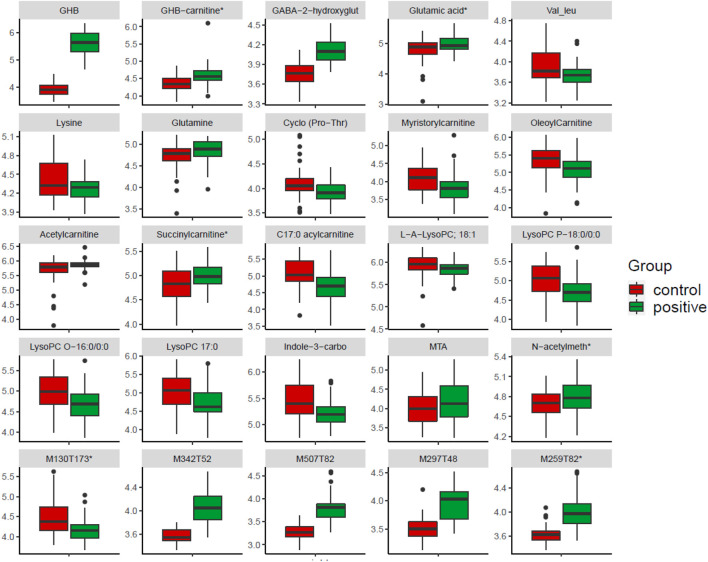
Boxplot of selected features showing up and down regulated metabolites in the GHB positive samples compared to the control group. Features marked with “*” means they have reported in controlled studies. The figure shows 25 features selected from Table 1, which includes features reported in controlled studies and interesting metabolites included in correlation network, and also unknowns with high VIP score. N-acetylmeth is N-acetylmethionine, Indole-3-carbo is indole-3-carboxaldehyde, GABA-2-hydroxyglut is GABA-2-hydroxyglutarate, which is tentatively identified.

Based on our combined feature selection strategy, 89 features were selected for further correlation network analysis (see next paragraph), and 77 of them show significant correlation with each other after FDR correction. Table 1 only includes the features with the top 20 highest VIPs values and top 20 highest correlation coefficients, also together with all the identified metabolites that match with in-house database and shows significance in at least one method (FC, VIP, and *q*-value). The remaining unknowns are shown in Supplementary Table S5. Twenty-five features of the features included in Table 1 are plotted in a box plot (Figure 5), to illustrate the difference of abundance between the control and positive group.

### GHB Impacts on Human Metabolism as Reflected by a Correlation Network

**FIGURE 6:**
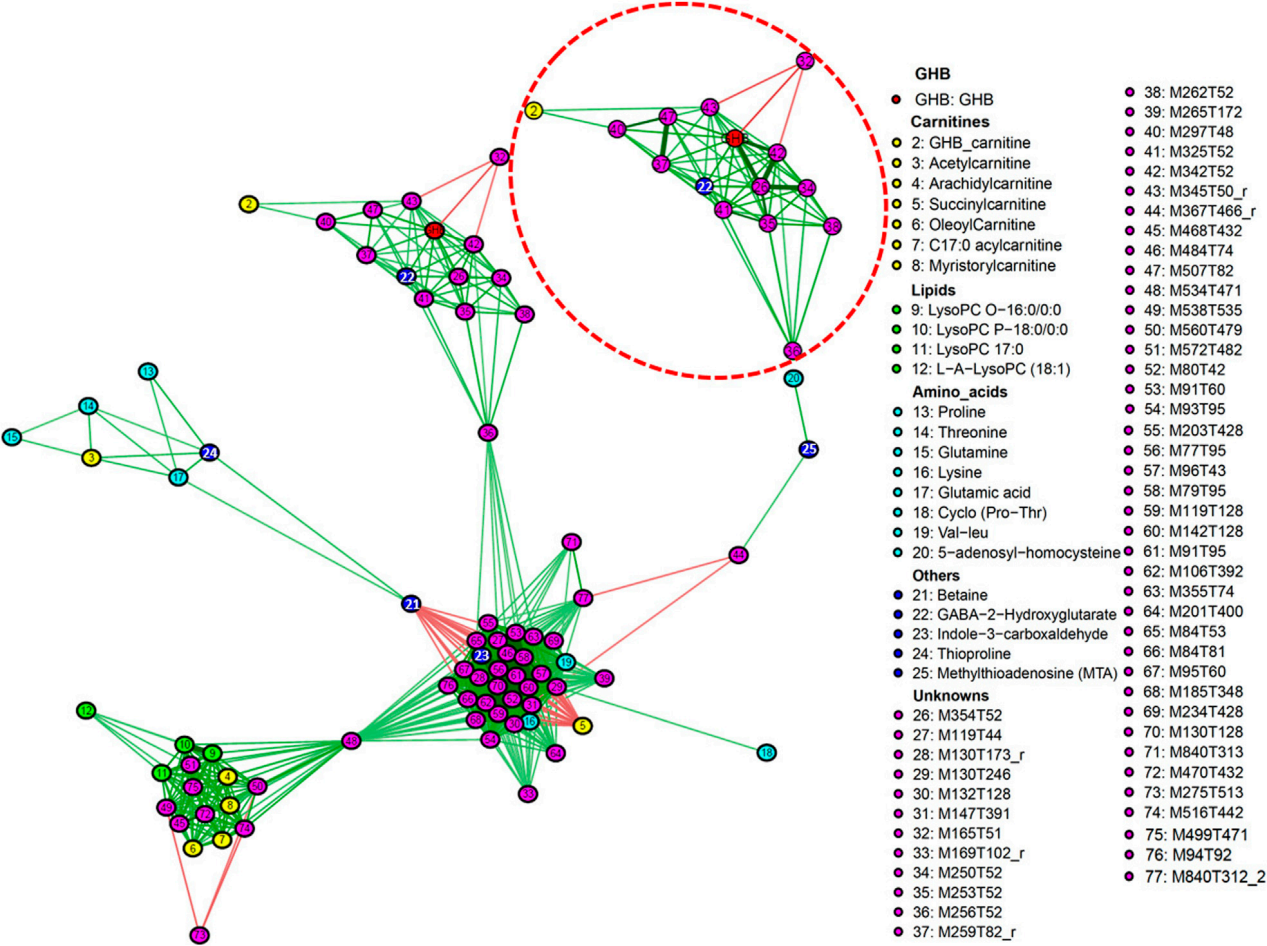
Correlation network of features associated with GHB intake. Edges represent correlations between features, edges in green mean positive correlations, edges in red mean negative correlation. Correlations between features were considered significant if FDR-corrected *q*-value < 0.1, 77 significant features are shown in this network. The subnetwork on the top is enlarged with a different scaling of edges to get more visible relations between these features. Only networks containing a minimum of three molecules are plotted.

The correlation network (CN) provides insights into the impact of GHB on metabolism and the interaction between the individual metabolites, this even though many features were not identified. Based on our combined feature selection strategy, 89 features were initially selected for further analysis. Of these 77 features are included in Figure 6, as only networks containing a minimum of three molecules were plotted. The CN highlighted the role of important features, and there are three main subnetworks shown in the plot. The highlighted subnetwork on the top shows the main features that correlate to GHB directly, e.g., M354T52 (#26), M250T52 (#34), M342T52 (#42), M259T82 (#37), M507T82 (#47), M234T52 (#22, possibly GABA-2-hydroxyglutarate), M345T50 (#43), M297T348 (#40), are positively correlated to GHB, while M165T51 (#32) is negatively correlated to GHB, M342T52 (#42), and M345T50 (#43). To show the correlation of features to GHB more clearly, we also zoomed in on the subnetwork that includes GHB with a different scaling of edges. We speculate that the features #26, #34, #42 might be direct adducts of GHB as the wider edges show a stronger correlation. The intensity of feature #32 is not high enough to be fragmented in DDA mode, while it is still interesting to notice that this feature is highly downregulated by GHB. GHB-carnitine (#2) is directly correlated to M297T48 (#40) and M345T50 (#43), and subsequently correlated to GHB. A strong correlation is found between feature #47 and #37, which indicates they may belong to the same compound as also noted previously. The subnetwork including GHB is connected to another subnetwork in the middle according to feature M256T52 (#36). Succinylcarnitine is negatively correlated to features including lysine, Indole-3-carboxaldehyde, and a number of unknowns (#27, 29, 46, 52, 57–60), and betaine is also negatively correlated to indole-3-carboxaldehyde, and a range of unknowns (#27, 31, 46, 52, 57–62). Top-left subnetwork contains acetylcarnitine, thioproline, and four amino acids (proline, glutamic acid, threonline, and glutamine), which are correlated to betaine and connected to the middle subnetwork. LysoPC O-16:0/0:0, LysoPC P-18:0/0:0, LysoPC (17:0), myristorylcarnitine, oleoylcarnitine, arachidylcarnitine, C17:0-acylcarnitine, and several knowns are included in the subnetwork bottom-left side, which shows that carnitines and lipids are closely related. It is found that mainly carnitines and lipids are included in this subnetwork, and they are connected to the middle subnetwork according to #48.

## Discussion

The main findings of the present study is the demonstration that it is indeed possible to use archived data normalized by NOMIS for identification of metabolites correlated to drug intake—here demonstrated with GHB using a combination of OPLS-DA, Pearson correlation and FDR *q*-value. In the data processing part, our results prove that the NOMIS normalization using multiple internal standards is a superior normalization method for retrospective analysis. The typical batch correction methods that are widely applied in metabolomics studies need a number of pooled quality control (QC) samples, while our data were efficiently normalized without using QC. Despite the large intensity variation in the raw data, the NOMIS can still take the analytical variation corresponding to sample preparation and ion source variation into account, and makes it possible to get good correlation between actual concentration and corrected peak areas.

As use of archived data for metabolite correlation is still in its infancy, we performed a range of advanced both statistical and machine learning methods to evaluate which performs the best. In general, OPLS-DA, Pearson correlation, and FDR *q*-values give the most valuable information when evaluating based on the number of prioritized metabolites found, that also have been reported in previous controlled studies (Steuer et al., 2019). These feature selection methods have also been used in previous metabolomics studies (Nielsen et al., 2016; Elmsjö et al., 2020; Jung et al., 2021), where meaningful results also were reported using these methods. Mass spectrometrory metabolome data analysis is complicated, since metabolites interact nonlinearly, and the data structures themselves are complex, especially when the study is retrospective and uncontrolled. Supervised machine learning has great potential in metabolomics research because of the ability to supply quantitative predictions (Jung et al., 2021). In this study, the machine learning model OPLS-DA gives better results than other machine learning models LASSO and random forest, while these models still provide compensatory information for feature selection that we could investigate in the future. Of important relevance is the combination of different feature selection methods to discover the potential biomarkers.

GHB-carnitine was first identified tentatively in urine samples in the study of Steuer et al. (2019), and further verified with standard in their latest study (Jung et al., 2021). Although GHB-carnitine was not detectable in serum in the study of Steuer et al. (2019), we routinely detected it using our analytical setup on the whole blood DUID cases. This is likely due to differences between our extraction or analytical methods or alternatively caused by the uncontrolled and potentially higher recreative consumption of GHB in the DUID cases. GHB-carnitine is the ester between GHB and carnitine. Carnitine is a small and highly polar zwitterionic compound that plays a critical role in energy metabolism and β-oxidation by facilitating transport of conjugated long chain fatty acids or more simple organic acids across the mitochondrial membrane (Bremer, 1983; Mo et al., 2014). Usually the synthesis of acylcarnitines proceed via an acyl SCoA intermediate, where the activated acyl group is then transferred to carnitine in a second step catalyzed by a carnitine acyltransferase. The current findings further suggest that whole blood GHB-carnitine could be a potential marker for exogenous GHB intake.

M259T82 shows particularly high significance in various statistics, and previously has been reported to be correlated to GHB intake even though it is still unknown without fragment pattern being provided (Steuer et al., 2019). The unknown features M507T82 also show very high significance being top 10 in all the models we applied, M259T82 and M507T82 might belong to the same compound due to the strong correlation in Pearson correlation (Figure 6) and also the same RT. A feature with similar mass to feature M234T52 has previously been identified tentatively as GHB-glutamate (Steuer et al., 2021). However, by comparison to a synthesized reference of GHB-glutamate we could not verify the identity in our case. Instead, we tentativly propose the feature as GABA-2-hydroxyglutarate (Supplementary Figure S12), which has the same mass as GHB-glutamate, but different RT and fragmentation pattern. GABA-2-hydroxyglutarate is an ester of GABA and 2-hydroxyglutaric acid, the latter which has been shown to be a prominent metabolite of GHB in mammals (Struys et al., 2006), and it indicates GHB intake with very high correlation. M507T82 or M259T82, and the tentative metabolite GABA-2-hydroxyglutarate could all be used as potential biomarkers for GHB intake, and it is worthwhile to further identify these unknown features.

Various other carnitine metabolites are either found to be up or down regulated by GHB intake, e.g., oleoylcarnitine that has also been reported in a previous GHB study (Steuer et al., 2021). In relation to GHB-carnitine, succinylcarnitine is perhaps the most relevant one as a supportive biomarker to GHB-carnitine. Succinylcarnitine, was also observed to be upregulated in previous controlled studies as a result of GHB administration (Steuer et al., 2019; Jarsiah et al., 2020). Furthermore, succinate is known to be formed from GHB via oxidation to succinic semialdehyde and then to succinate, which then ultimately can enter into the citric acid cycle as an energy metabolite (Zhang et al., 2009). As succinate previously also have been reported to increase on GHB consumption, it seems likely that excess of succinate is diverted into succinyl SCoA and then finally succinylcarnitine following GHB intake. Increased levels of the two metabolites GHB and succinyl carnitine combined accordingly may more strongly support exogenous GHB intake. This together with the regulation of other strongly regulated metabolites we will need to verify in a controlled clinical study, in which the detection window of the relevant component can also be assessed.

In previous studies, several GHB-related acids have been reported in blood plasma and urine, such as glycolic acid, 3,4-DHB, 2,4-dihydroxybutyric acid, GA and some organic acids from the TCA cycle (Küting et al., 2021). None of these metabolites are found in our study as these acidic metabolites are mostly analyzed in negative mode, whereas only positive mode was used in this study. Therefore analysis in negative mode could be carried out in the future. In addition, three other unknowns, namely M127T391, M367T466, M169T102 that previously have been reported to be correlated to GHB intake (Steuer et al., 2019), are also detected in our work (Table 1). All these unknown features show significant effects in at least one of applied methods. These previously reported unknowns also validate our approach although they may not be direct GHB biomarkers.

To make a better understanding of the impact of GHB on human endogenous metabolism, a correlation network (CN) using all selected GHB-related metabolites was also constructed. The CN also reveals potential interaction or co-regulation between non-GHB features potentially revealing larger or more general metabolic impact on groups of related metabolites (Figure 4). From the Figure 4 it is evident that GHB impacts on the metabolism of many carnitines, lipids, amino acids, a range of unknowns as well as clustering of similar or biochemically related compounds further strengthens the evidence and insight. As previously mentioned, acylcarnitines mainly functions as entities for transporting organic acids into the mitochondria for oxidative metabolism (Skulachev, 1998; Alves et al., 2009). Increased levels of these compounds usually reflect increased levels of their immediate precursors, e.g., the increased level of acetyl carnitine reflects an increase or surplus of acetylSCoA following GHB ingestion (Rubaltelli et al., 1987). Why we observe a build-up of acetylcarnitine following GHB ingestion is unknown, particularly as a range of long chain acylcarnitines as well as lysoPC are down-regulated indicating that lipid metabolism more generally is impeded. A high upregulation of lysoPCs and carnitines in the brain of mice following acute GHB ingestion is demonstrated in the Study of Luca et al. (2014), while they did not see the same regulation in the liver of the mice. Significant regulation of these metabolites are found in blood in our study, though the regulation is opposite to what is observed in the cortex of mice, but it may suggest a role for these metabolites following GHB intake that perhaps is not directly involved in energy metabolism (Luca et al., 2014). Based on the CN it however appears that acetylcarnitine is more strongly correlated to several amino acids, e.g., glutamate, glutamine, proline and threonine, and as these can serve as metabolic fuel generating acetylSCoA, the increased acetylcarnitine may simply reflect an increased energy dependence on catabolism of these amino acids perhaps as substitution for the retarded lipid metabolism. In the CN, we could also find that glutamate, proline, glutamine and acetylcarnitine are grouped together on the left, and as they are metabolites of each other and accordingly biochemically connected as reported (Tapiero et al., 2002; Susanna et al., 2010), it further validates the outcome of our method and analysis. It can be mentioned that GHB intake also in previous studies have been correlated to increased levels of some of these amino acids (Steuer et al., 2021), where, e.g., glutamic acid is identified with a relative high VIP score (1.29). Several studies have furthermore shown effects of GHB on glutamate release (Ferraro et al., 2001; Castelli et al., 2003), and these results also suggest that the effects of GHB on glutamate release might be mediated by GHB receptors and GABA_B_ receptors. Proline and lysine have also been reported to be up and down regulated respectively by GHB exposure in rat, which is consistent with our results, and it could also be an additional validation of our method (Seo et al., 2018). Finally, we also found some amino acids not previously reported to be regulated by GHB intake, such as homocysteine, threonine, Phe-Val and cyclo (Pro-Thr). These metabolites provides supplementary information for the GHB metabolism interpretation and used as second targets of methods to elucidate the metabolic impact.

In relation to use of the features as discriminative biomarkers for GHB consumption, it is at the present stage difficult to evaluate whether the obtained biomarkers of GHB intake will be sufficiently strong and also persists long enough *in vivo* to be useful. We however, note that several of the presumptive direct metabolites of GHB are 2–3 fold upregulated and furthermore are highly significant, so potentially they are. Even more if combined with further known metabolites from other recently published studies. Furthermore, based on our machine learning models we indeed can discriminate presumed GHB users from non-users with more than 80% accuracy, e.g., using the OPLS-DA model, we get more than 80% accuracy in average for classification of groups indicating that discrimination with some certainty already at the present stage is possible. In the future with controlled follow-up studies, we will most likely be able to get even more robust data and accordingly accurate discriminations.

An increasing number of laboratories use UHPLC-HRMS routinely for screening of biological samples for different metabolites or exogenous compounds leading to a huge amount of data of potential high value. As conducting clinical studies is expensive and ethical problems also arise particular when the studies involve new and untested drugs of abuse, a data set from a controlled cohort is not always easily available. Retrospective metabolomics studies, e.g., mining archived data from routine screenings gives a unique opportunity to access such data at almost no cost. This obviously is important within forensic science, where many new illegal drugs constantly are appearing, and little is known about their metabolites as well as their potential impact on human metabolism. Apart from solving this forensic toxicology issue, the use of archived data furthermore allows access to larger samples sizes than usual in controlled studies.

Our study demonstrates the power of this approach by initially detecting a range of potential biomarkers of GHB consumption as well as reveal how GHB intake further regulate endogenous metabolism. Many of the discoveries being validated by comparison to the literature. However, due to the uncontrolled population in this and future similar studies, there are obviously also several potential confounders such as the unknown interval from intake to blood sampling and dose, the varying metabolic rate between individuals, diet, the activity level, and the tolerance to continuous use of GHB (or another drug), Furthermore, the setup we use can also be improved, e.g., by inclusion of further QC samples to improve normalization as well as it is advisable also to perform routine analysis of the samples in the negative mode in future to include metabolite coverage. In the current study, we tried carefully to select a control group that matched the GHB positive group with regard to additional drug intake. This however cannot be perfect and should be kept in mind during data analysis. Despite various challenges corresponding to the cohort in this study, our findings and validation to the literature prove that it is still feasible to utilize UHPLC-HRMS screening data from long term forensic studies.

## Conclusion

A general workflow is developed to carry out metabolomics studies on archived HRMS data from routine UHPLC-TOF screenings. The principle is demonstrated by using analytical data from a selection of GHB positive and matched control cases and validated by comparison of the results to those observed in controlled GHB studies. Generally, we rediscover a range of previously reported direct GHB metabolites, as well as we observe regulation of endogenous metabolites both some previously known, but also novel findings of potential biochemical relevance. We apply data acquired over a quite extensive time frame (6 years) indicating the robustness of the method. The study gives a further proof-of-principle on use of archived data when ordinary human data are unavailable, and paves the way for both a direct and simple elucidation of metabolites of new legal or illegal drugs as well as open up for large scale metabolomics studies for more general use of archived data in the future. This can be from local databases as in this case or, e.g., more big-data approach to disease prevention and detection using more extensive data sets or blood samples. Obviously, a range of cofounders will always exist in such datasets due to the uncontrolled approach and this needs to be taken into consideration when evaluating the results. The use of archived data however has so many advantages including being significantly cheaper than performing clinical studies, that the approach merits further use and we strongly believe that it in the future will become routine in laboratories applying such screening procedures.

## Data Availability

The original contributions presented in the study are included in the article/Supplementary Material, further inquiries can be directed to the corresponding authors.
