# Supplementary material for: A Retrospective Metabolomics Analysis of Gamma-Hydroxybutyrate in Humans: New Potential Markers and Changes in Metabolism Related to GHB Consumption
Source: Front Pharmacol. 2022 Mar 3;13:816376. doi: 10.3389/fphar.2022.816376 (PMC8927817; doi:10.3389/fphar.2022.816376)
Supplement: Supplementary file 1 [file DataSheet1.zip › Data Sheet 1/Raw data and R code/correlation and network/Rplot01.pdf]

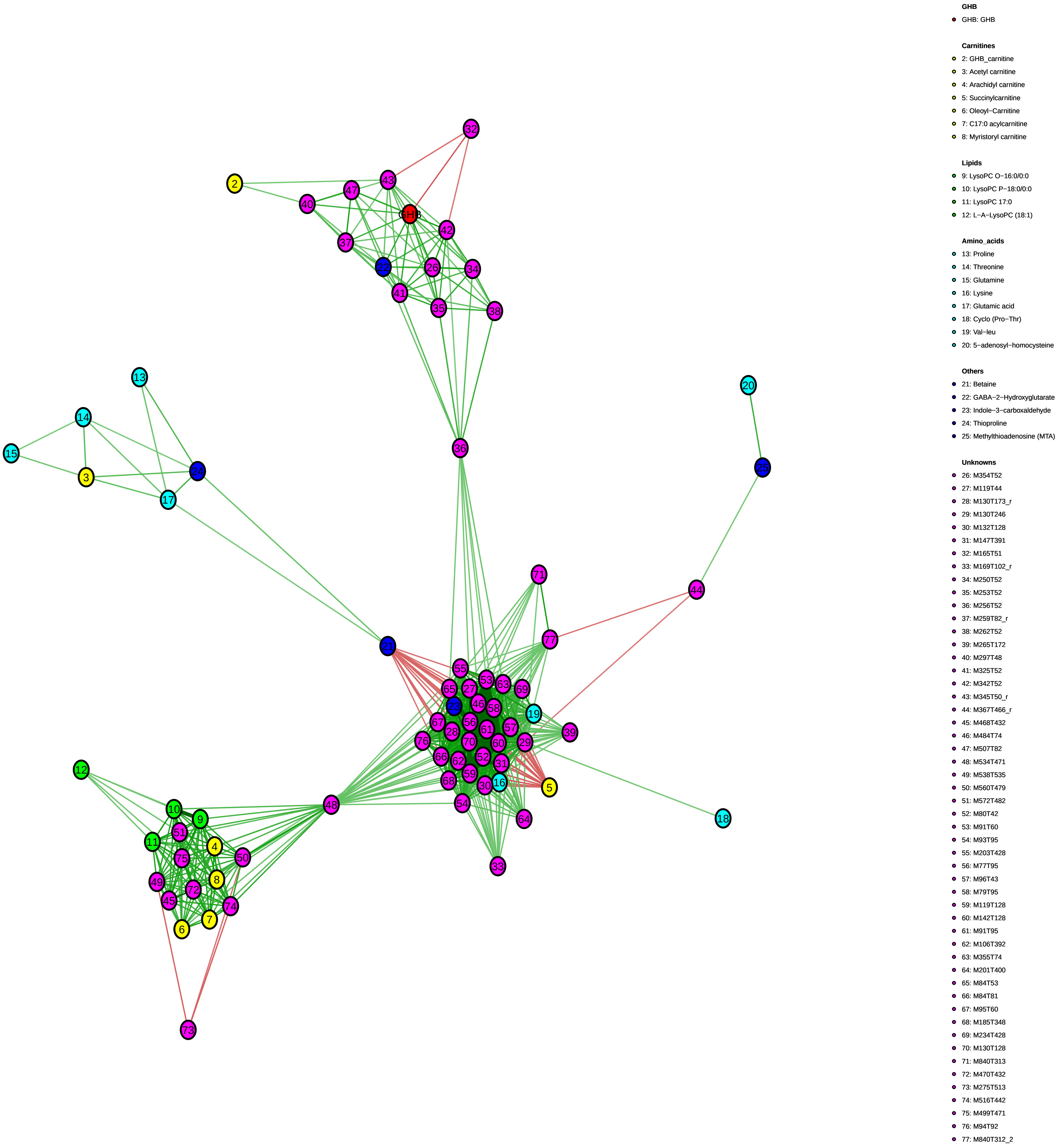
GHB
GHB: GHB
Carnitines
2: GHB_carnitine
3: Acetyl carnitine
4: Arachidyl carnitine
5: Succinylcarnitine
6: Oleoyl−Carnitine
7: C17:0 acylcarnitine
8: Myristoryl carnitine
Lipids
9: LysoPC O−16:0/0:0
10: LysoPC P−18:0/0:0
11: LysoPC 17:0
12: L−A−LysoPC (18:1)
Amino_acids
13: Proline
14: Threonine
15: Glutamine
16: Lysine
17: Glutamic acid
18: Cyclo (Pro−Thr)
19: Val−leu
20: 5−adenosyl−homocysteine
Others
21: Betaine
22: GABA−2−Hydroxyglutarate
23: Indole−3−carboxaldehyde
24: Thioproline
25: Methylthioadenosine (MTA)
Unknowns
26: M354T52
27: M119T44
28: M130T173_r
29: M130T246
30: M132T128
31: M147T391
32: M165T51
33: M169T102_r
34: M250T52
35: M253T52
36: M256T52
37: M259T82_r
38: M262T52
39: M265T172
40: M297T48
41: M325T52
42: M342T52
43: M345T50_r
44: M367T466_r
45: M468T432
46: M484T74
47: M507T82
48: M534T471
49: M538T535
50: M560T479
51: M572T482
52: M80T42
53: M91T60
54: M93T95
55: M203T428
56: M77T95
57: M96T43
58: M79T95
59: M119T128
60: M142T128
61: M91T95
62: M106T392
63: M355T74
64: M201T400
65: M84T53
66: M84T81
67: M95T60
68: M185T348
69: M234T428
70: M130T128
71: M840T313
72: M470T432
73: M275T513
74: M516T442
75: M499T471
76: M94T92
77: M840T312_2
