# Supplementary figures and images for: A Retrospective Metabolomics Analysis of Gamma-Hydroxybutyrate in Humans: New Potential Markers and Changes in Metabolism Related to GHB Consumption
Source: Front Pharmacol. 2022 Mar 3;13:816376. doi: 10.3389/fphar.2022.816376 (PMC8927817; doi:10.3389/fphar.2022.816376)

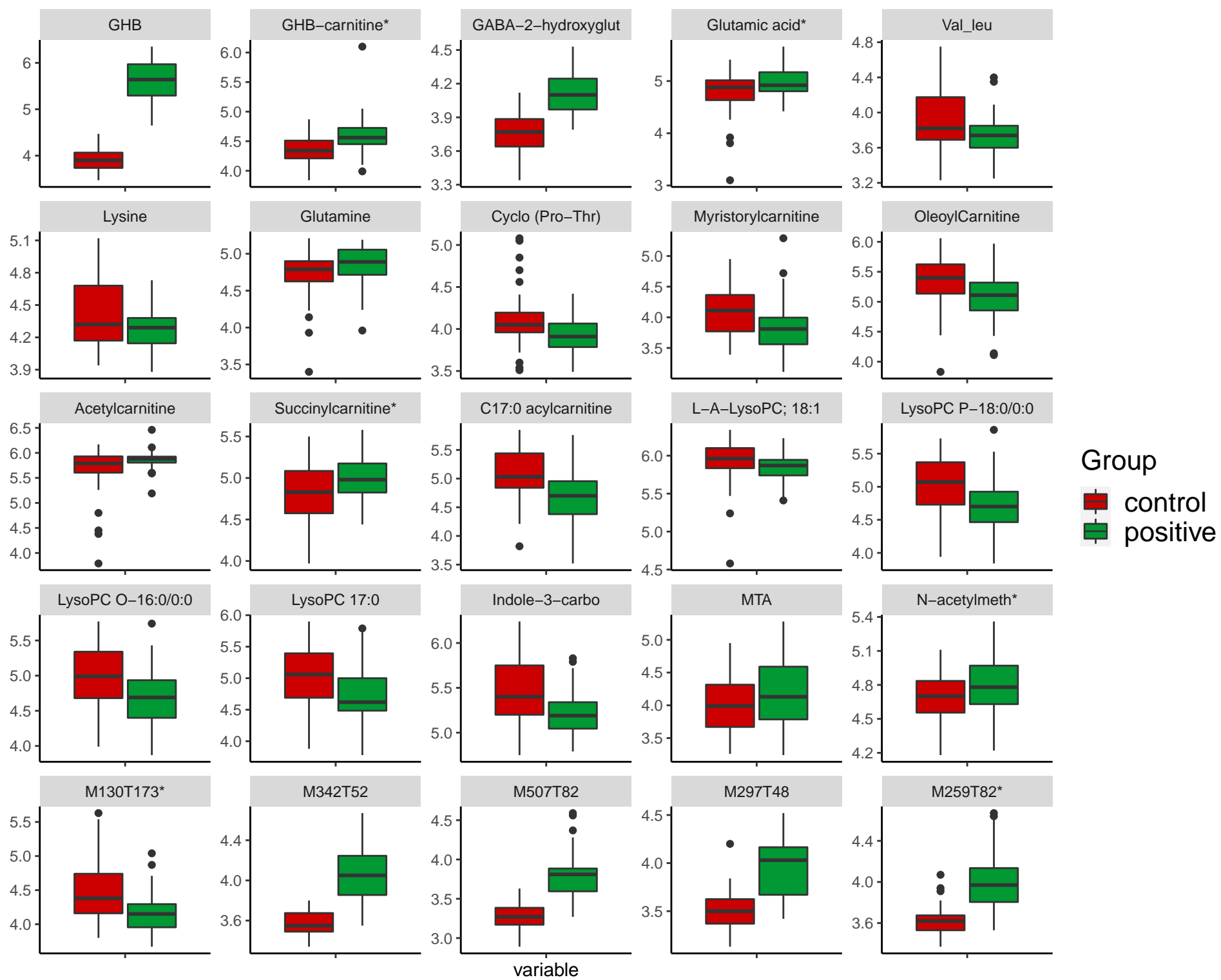

Supplement: Supplementary file 1 [file DataSheet1.zip › Data Sheet 1/Raw data and R code/boxplot/boxplot.pdf]

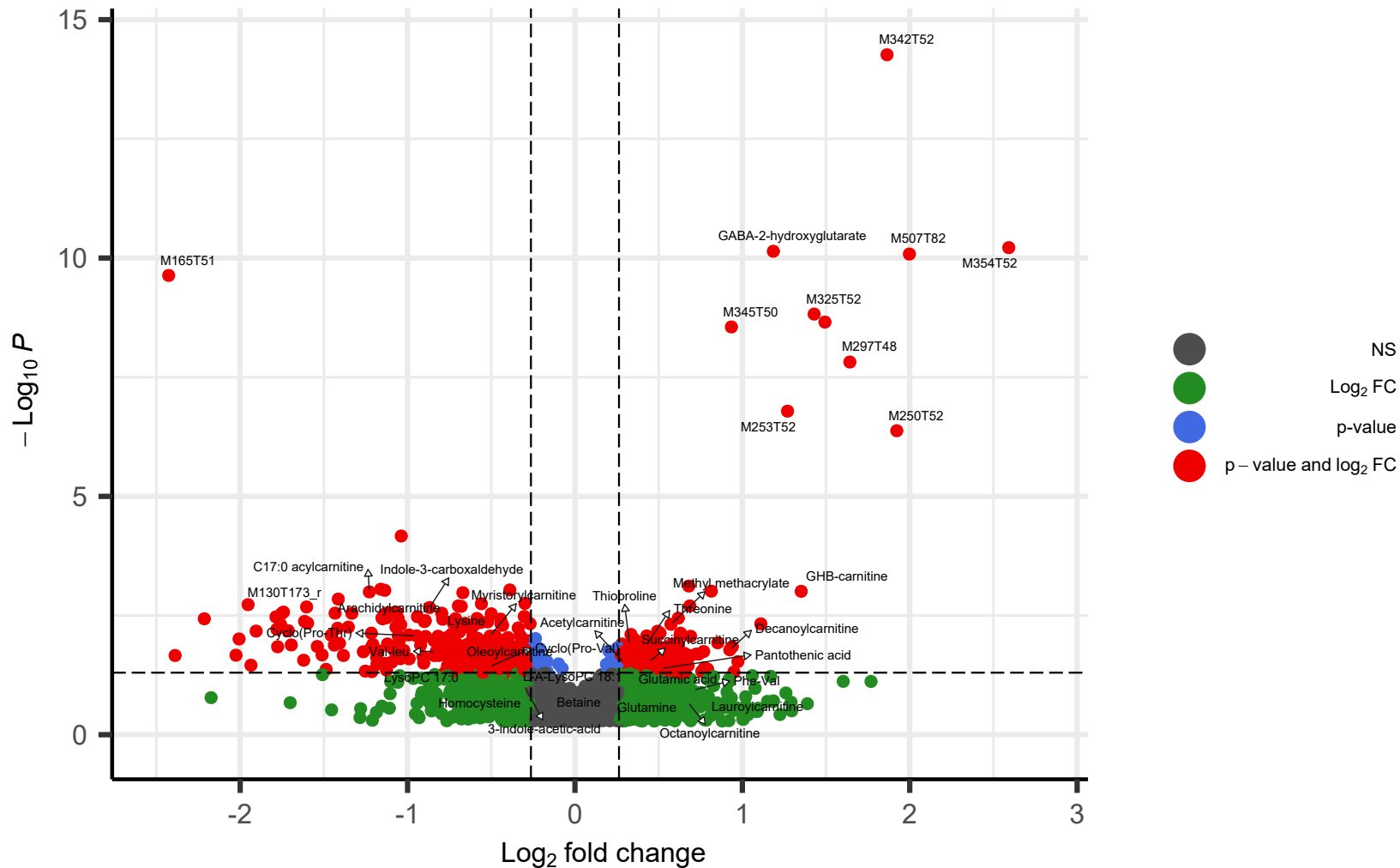

Supplement: Supplementary file 1 [file DataSheet1.zip › Data Sheet 1/Raw data and R code/volcano plot/volcano-plot_2022_0125.pdf]
