# Supplementary material for: A Retrospective Metabolomics Analysis of Gamma-Hydroxybutyrate in Humans: New Potential Markers and Changes in Metabolism Related to GHB Consumption
Source: Front Pharmacol. 2022 Mar 3;13:816376. doi: 10.3389/fphar.2022.816376 (PMC8927817; doi:10.3389/fphar.2022.816376)
Supplement: Supplementary file 2 [file DataSheet1.docx]

**Supporting information**

# A Retrospective Metabolomics Analysis of Gamma‐hydroxybutyrate (GHB) in Humans: New Potential Markers and Changes in Metabolism Related to GHB Consumption

Tingting Wang^1^, Kirstine L. Nielsen^1^, Kim Frisch^1^, Johan K. Lassen^2^, Camilla B. Nielsen^1^, Charlotte U. Andersen^1^, Palle Villesen^2^, Mette F. Andreasen^1^, Jørgen B. Hasselstrøm^1^, Mogens Johannsen^1^

1. Department of Forensic Medicine, Section for Forensic Chemistry, Aarhus University, Palle Juul- Jensens Boulevard 99, DK-8200 Aarhus N, Denmark

2. Bioinformatics Research Centre, Aarhus University, C. F. Møllers Allé 8, 8000, Aarhus C, Denmark

-----------------------------------------

* Corresponding author: Mogens Johannsen; Tingting Wang

E-mail address: [mj@forens.au.dk](mailto:mj@forens.au.dk); [ting@forens.au.dk](mailto:ting@forens.au.dk)

# Supporting information

# Tables

Table S1. List of drugs identified and quantified in all samples used for data analysis

Table S2. Information of GHB positive and negative case

Table S3. Full parameters for peak extraction using XCMS based approach

Table S4. Results summary of all the features by different methods (shown in excel sheet)

Table S5. Unknown features found to discriminate GHB positive samples from control samples

Table S6. Fragment pattern of tentatively identified metabolites or unknowns

Table S7. Output from XCMS (shown in excel sheet)

Table S8. Peak integration accuracy of GHB (shown in excel sheet)

Table S9. Z-normalization of data (shown in excel sheet)

Table S10. NOMIS-normalization of data (shown in excel sheet)

# Figures

Figure. S1. Retention time deviation of all GHB samples

Figure. S2. Variation of internal standards in all the studied samples

Figure S3. Accuracy of GHB peak area integrated by XCMS

Figure S4. PCA plot of GHB positive and control samples

Figure. S5. Spectrum of GHB positive sample spiked with GHB-glutamate

Figure S6. Synthesis of tribenzyloxy GHB-glutamate

Figure S7. ^1^H NMR of tribenzyloxy GHB-glutamate

Figure S8. ^13^C NMR of tribenzyloxy GHB-glutamate

Figure S9. Synthesis of GHB-glutamate

Figure S10. ^1^H NMR of GHB-glutamate

Figure S11. ^13^C NMR of GHB-glutamate

Figure S12. Proposed structure of feature M234T52 and possible pathway

# Others

Method and material of GHB glutamate synthesis

| Drugs (Metabolites) | GHB positive | Control |
| --- | --- | --- |
| GHB | 51 | 0 |
| Cocain | 33 | 22 |
| Tetrahydrocannabinol (THC) | 22 | 25 |
| Amfetamin | 20 | 23 |
| Nordazepam | 14 | 12 |
| Ketamin | 14 | 8 |
| Diazepam | 14 | 13 |
| Alprazolam | 12 | 8 |
| Clonazepam | 9 | 14 |
| Oxazepam | 5 | 2 |
| Temazepam | 5 | 5 |
| 3,4-methylenedioxy-methamphetamine (MDMA) | 3 | 8 |
| 2,3-Methylenedioxyamphetamine (MDA) | 3 | 6 |
| Methylphenidat | 2 | 2 |
| Metamfetamin | 2 | 1 |
| 11-Nor-9-carboxy-Δ9-tetrahydrocannabinol (THC-COOH) | 1 | 3 |
| Bromazepam | 1 | 0 |
| Fentanyl | 1 | 1 |
| N-[(1S)-1-(aminocarbonyl)-2-methylpropyl]-1-[(4-fluorophenyl)methyl]-1H-indazole-3-carboxamide (AB-FUBINACA) | 1 | 0 |
| Methadon | 1 | 3 |
| Lysergic acid diethylamide (LSD) | 0 | 1 |
| Nitrazepam | 0 | 2 |
| Morphin | 0 | 4 |
| Buprenorphin | 0 | 3 |
| Oxycodon | 0 | 4 |
| Delorazepam | 0 | 1 |
| Diclazepam | 0 | 1 |

**Table** S1. List of drugs identified and quantified in all samples used for data analysis

Notes: the data both for control group and GHB positive group samples were all collected from 2015 to 2020, the details of data collection time can be found in SI in excel sheet Table S10.

**Table** S2. Information of GHB positive and negative case.

| Positive samples and collection year | | GHB (mg/kg) | Control samples and collection year | |
| --- | --- | --- | --- | --- |
| TX1 | 2015 | 28.00 | TXC1 | 2015 |
| TX2 | 2016 | 83.00 | TXC2 | 2015 |
| TX3 | 2016 | 10.00 | TXC3 | 2015 |
| TX4 | 2017 | 73.00 | TXC4 | 2016 |
| TX5 | 2017 | 140.00 | TXC5 | 2016 |
| TX6 | 2017 | 130.00 | TXC6 | 2016 |
| TX7 | 2017 | 180.00 | TXC7 | 2016 |
| TX8 | 2017 | 44.13 | TXC8 | 2016 |
| TX9 | 2017 | 24.39 | TXC9 | 2016 |
| TX10 | 2018 | 98.12 | TXC10 | 2017 |
| TX11 | 2018 | 30.45 | TXC11 | 2017 |
| TX12 | 2018 | 173.03 | TXC12 | 2017 |
| TX13 | 2018 | 34.73 | TXC13 | 2017 |
| TX14 | 2018 | 30.06 | TXC14 | 2018 |
| TX15 | 2018 | 32.51 | TXC15 | 2018 |
| TX16 | 2018 | 104.75 | TXC16 | 2018 |
| TX17 | 2018 | 119.85 | TXC17 | 2018 |
| TX18 | 2018 | 151.11 | TXC18 | 2018 |
| TX19 | 2018 | 45.68 | TXC19 | 2018 |
| TX20 | 2018 | 23.97 | TXC20 | 2018 |
| TX21 | 2019 | 31.76 | TXC21 | 2018 |
| TX22 | 2019 | 20.44 | TXC22 | 2018 |
| TX23 | 2019 | 37.10 | TXC23 | 2018 |
| TX24 | 2019 | 36.91 | TXC24 | 2019 |
| TX25 | 2019 | 13.70 | TXC25 | 2019 |
| TX26 | 2019 | 18.26 | TXC26 | 2019 |
| TX27 | 2019 | 85.37 | TXC27 | 2019 |
| TX28 | 2019 | 103.02 | TXC28 | 2019 |
| TX29 | 2019 | 35.21 | TXC29 | 2019 |
| TX30 | 2019 | 181.40 | TXC30 | 2019 |
| TX31 | 2019 | 148.84 | TXC31 | 2019 |
| TX32 | 2019 | 67.15 | TXC32 | 2019 |
| TX33 | 2019 | 189.14 | TXC33 | 2020 |
| TX34 | 2019 | 51.88 | TXC34 | 2020 |
| TX35 | 2019 | 143.68 | TXC35 | 2020 |
| TX36 | 2019 | 231.53 | TXC36 | 2020 |
| TX37 | 2019 | 17.58 | TXC37 | 2020 |
| TX38 | 2020 | 153.47 | TXC38 | 2020 |
| TX39 | 2020 | 13.36 | TXC39 | 2020 |
| TX40 | 2020 | 144.32 | TXC40 | 2020 |
| TX41 | 2020 | 47.30 | TXC41 | 2020 |
| TX42 | 2020 | 48.66 | TXC42 | 2020 |
| TX43 | 2020 | 125.34 | TXC43 | 2020 |
| TX44 | 2020 | 94.64 | TXC44 | 2020 |
| TX45 | 2020 | 65.06 | TXC45 | 2020 |
| TX46 | 2020 | 87.78 | TXC46 | 2020 |
| TX47 | 2020 | 71.25 | TXC47 | 2020 |
| TX48 | 2020 | 102.27 | TXC48 | 2020 |
| TX49 | 2020 | 94.12 | TXC49 | 2020 |
| TX50 | 2020 | 151.65 | TXC50 | 2020 |
| TX51 | 2020 | 78.17 | TXC51 | 2020 |

| **Table** S3. Full parameters for peak extraction using XCMS based approach | | |
| --- | --- | --- |
| **Step** | **Parameter** | **Value** |
| Xcmset | Extraction method for peaks detection | centWave |
|  | ppm | 15 |
|  | peakwidth | 5-20 |
|  | mzdiff | 0.01 |
|  | snthresh | 6 |
|  | prefilter | 3,500 |
|  | mslevel | 1 |
| Group - 1 | Method to use for grouping | PeakDensity |
|  | bw | 10 |
|  | minFraction | 0.5 |
|  | binSize | 0.025 |
|  | maxFeatures | 100 |
| Retcor | Method to use for retention time correction | peakgroups |
|  | smooth | loess |
|  | minFraction | 0.5 |
| Group - 2 | Method to use for grouping | PeakDensity |
|  | bw | 2.5 |
|  | mzwid | 0.01 |
|  | minFraction | 0.5 |
|  | maxFeatures | 100 |
| Camera | perfwhm | 0.3 |
|  | mzabs | 0.001 |
|  | ppm | 3 |
|  | intval | into |
|  | maxcharg | 8 |
|  | minfrac | 0.2 |
|  | maxiso | 5 |

Note: bw is related to retention time, it represents the standard deviation of the gaussian metapeak that group peaks together. minFraction: a group must be found in at least minFraction*n samples, with n=number of samples for each class of samples. A minFraction=0.5 corresponds to 50%. More explanation of these xcms parameters could be found here https://bioconductor.org/packages/release/bioc/manuals/xcms/man/xcms.pdf.

**Table** S5. Unknown features found to discriminate GHB positive samples from control samples.

| Name | Annotation | rt_min | Dir | FC | m/z | VIP | P | FDR q-value | Lasso | %IncMSE | MDA | PC |
| --- | --- | --- | --- | --- | --- | --- | --- | --- | --- | --- | --- | --- |
| M132T128 | Unknown | 2.14 | ↓ | 0.45 | 132.0807 | 2.05 | <0.001 | <0.001 | 0 | -1.33 | 1.42 | -0.38 |
| M534T471 | Unknown | 7.85 | ↓ | 0.68 | 534.2952 | 1.90 | <0.001 | 0.002 | 0 | 1.00 | 0.00 | -0.37 |
| M578T70 | Unknown | 1.16 | ↑ | 1.40 | 578.2972 | 1.43 | <0.001 | 0.012 | 0 | 0.96 | 0.98 | 0.37 |
| M203T428 | Unknown | 7.14 | ↓ | 0.48 | 203.1066 | 2.06 | <0.001 | 0.003 | 0 | 0.86 | 0.85 | -0.37 |
| M275T513 | Unknown | 8.54 | ↑ | 1.41 | 275.2577 | 1.77 | <0.001 | 0.008 | 0 | -0.92 | -1.00 | 0.37 |
| M470T432 | Unknown | 7.19 | ↓ | 0.61 | 470.3141 | 1.97 | <0.001 | 0.004 | 0 | 1.37 | 1.00 | 0.37 |
| M215T75_1 | Unknown | 1.26 | ↓ | 0.79 | 215.1387 | 1.28 | 0.005 | 0.027 | 0 | 2.08 | 1.00 | 0.37 |
| M198T159 | Unknown | 2.64 | ↓ | 0.74 | 198.1315 | 1.42 | <0.001 | 0.005 | 0 | 1.09 | 1.56 | 0.36 |
| M516T442 | Unknown | 7.37 | ↓ | 0.58 | 516.3057 | 2.07 | <0.001 | 0.003 | 0 | -1.19 | -1.42 | 0.36 |
| M929T287 | Unknown | 4.78 | ↑ | 1.38 | 928.6994 | 1.25 | 0.001 | 0.013 | 0.021 | 1.31 | -1.00 | 0.36 |
| M385T516 | Unknown | 8.60 | ↑ | 1.55 | 385.1541 | 1.71 | <0.001 | 0.007 | 0 | -1.32 | 2.10 | 0.36 |
| M707T279_2 | Unknown | 4.65 | ↑ | 1.33 | 707.3367 | 1.26 | 0.002 | 0.017 | 0 | -1.19 | 0.02 | 0.36 |
| M130T246 | Unknown | 4.11 | ↓ | 0.49 | 130.0651 | 2.20 | <0.001 | <0.001 | 0 | 1.00 | 1.89 | -0.36 |
| M310T26 | Unknown | 0.43 | ↑ | 1.61 | 310.1666 | 1.75 | <0.001 | 0.002 | 0 | -0.29 | 1.00 | 0.36 |
| M499T471 | Unknown | 7.85 | ↓ | 0.67 | 499.3474 | 1.78 | <0.001 | 0.004 | 0 | 0.89 | 1.00 | 0.35 |
| M94T92 | Unknown | 1.53 | ↓ | 0.63 | 94.0649 | 1.94 | <0.001 | 0.001 | 0 | -0.94 | 0.00 | 0.35 |
| M830T303_2 | Unknown | 5.04 | ↓ | 0.74 | 830.3230 | 1.52 | <0.001 | 0.005 | 0 | -0.99 | 0.35 | 0.35 |
| M840T312_2 | Unknown | 5.21 | ↓ | 0.80 | 840.4558 | 1.17 | <0.001 | 0.008 | 0 | 1.40 | -0.51 | 0.35 |
| M560T479 | Unknown | 7.99 | ↓ | 0.67 | 560.3109 | 2.05 | <0.001 | 0.004 | 0 | 0.77 | 1.49 | -0.35 |
| M468T432 | Unknown | 7.19 | ↓ | 0.54 | 468.3089 | 2.19 | <0.001 | 0.004 | 0 | 1.69 | 1.35 | -0.35 |
| M675T274_3 | Unknown | 4.56 | ↑ | 1.41 | 675.1029 | 1.71 | <0.001 | 0.007 | 0 | 0.31 | 1.00 | 0.35 |
| M142T128 | Unknown | 2.14 | ↓ | 0.30 | 142.0649 | 2.28 | 0.0001 | 0.003 | 0 | -1.10 | 1.39 | 0.31 |
| M91T95 | Unknown | 1.00 | ↓ | 0.25 | 91.0542 | 2.23 | 0.0005 | 0.004 | 0 | -1.00 | 1.42 | 0.25 |
| M77T95 | Fragment of M93T95 | 1.58 | ↓ | 0.33 | 77.0384 | 2.27 | <0.001 | 0.004 | 0 | -1.00 | 1.00 | -0.30 |
| M106T392 | Unknown | 6.53 | ↓ | 0.31 | 106.0733 | 2.24 | <0.001 | 0.007 | 0 | 1.04 | 0.00 | 0.27 |
| M91T60 | Unknown | 1.00 | ↓ | 0.25 | 91.05416 | 2.23 | <0.001 | 0.010 | 0 | -1.00 | 1.42 | -0.25 |
| M265T172 | Unknown | 2.86 | ↓ | 0.46 | 265.0503 | 2.23 | 0.011 | 0.044 | 0 | 1.11 | 0.00 | -0.26 |
| M119T44 | Unknown | 0.73 | ↓ | 0.30 | 119.0351 | 2.23 | <0.001 | 0.005 | 0 | -1.00 | 1.00 | -0.28 |
| M200T23 | Unknown | 0.38 | ↑ | 1.96 | 200.1281 | 2.23 | 0.005 | 0.029 | 0 | -0.88 | 1.00 | 0.30 |
| M355T74 | Unknown | 1.24 | ↓ | 0.26 | 355.0737 | 2.22 | 0.007 | 0.035 | 0 | 0.57 | 0.00 | 0.22 |
| M201T400 | Unknown | 6.67 | ↓ | 0.54 | 201.1277 | 2.22 | <0.001 | 0.004 | 0 | 0.96 | -0.58 | 0.32 |
| M84T53 | Unknown | 0.88 | ↓ | 0.39 | 84.0444 | 2.22 | <0.001 | 0.006 | 0 | -0.91 | 0.00 | 0.28 |
| M484T74 | Unknown | 1.23 | ↓ | 0.33 | 484.1164 | 2.21 | <0.001 | 0.005 | 0 | 0.76 | -1.00 | -0.27 |
| M572T482 | Unknown | 8.03 | ↓ | 0.59 | 572.3711 | 2.20 | <0.001 | 0.009 | 0 | 1.00 | 1.00 | -0.31 |
| M84T81 | Unknown | 1.34 | ↓ | 0.40 | 84.0807 | 2.18 | <0.001 | 0.003 | 0 | 0.00 | 0.00 | 0.30 |
| M185T348 | Unknown | 5.80 | ↓ | 0.19 | 185.1538 | 2.13 | 0.003 | 0.022 | 0 | -1.00 | 1.00 | 0.21 |
| M234T428 | Unknown | 7.14 | ↓ | 0.37 | 234.1854 | 2.12 | 0.001 | 0.013 | 0 | -1.00 | -1.00 | 0.32 |
| M130T128 | Unknown | 2.14 | ↓ | 0.45 | 130.0651 | 2.11 | <0.001 | <0.001 | 0 | -1.00 | 0.00 | 0.34 |
| M147T391 | Unknown | 6.52 | ↓ | 0.48 | 147.0652 | 1.87 | <0.001 | 0.004 | 0 | 0.74 | 0.00 | -0.27 |
| M743T284_2 | Unknown | 4.74 | ↑ | 1.53 | 742.7594 | 1.67 | 0.001 | 0.013 | 0 | 1.12 | 1.27 | 0.35 |
| M95T60 | Unknown | 1.00 | ↓ | 0.29 | 95.0491 | 2.14 | <0.001 | 0.006 | 0 | 0.006 | -0.99 | 0.28 |

Note: Predicted by random forest, Pearson correlation, LASSO, and OPLS VIP-Scores, it is an extension of Table 1, as described in the features selection part. %IncMSE, percentage increase of the mean squared error. MDA, mean decrease accuracy. Dir, direction of regulation by GHB. PC, Pearson correlation coefficients.

**Table** S6. Fragment pattern of tentatively identified metabolites or unknowns

|  | ID | m/z | Fragments and abundance | Formula | Structures | Ident level |
| --- | --- | --- | --- | --- | --- | --- |
| 1 | M507T82 | 507.1547 | 271.0649 (100), 259.0792 (94.9) |  | Unknown |  |
| 2 | M248T57 | 248.1490 | 85.0286 (100)  144.1057 (11) | C_11_H_21_NO_5_ | GHB-carnitine | 1 |
| 3 | M192T140 | 192.0689 | 144.0658 (100), 150.0576(66.9), 174.0575 (21.1) | C_7_H_13_NO_3_S | N-acetylmethionine | 1 |
| 4 | M101T88 | 101.0597 | 85.0285  69.0335 | C_5_H_8_O_2_ | Methyl methacrylate | 2 |
| 5 | M675T274_3 | 675.1029 | 331.0678 (16.0)  478.7138 (37.1) |  | Unknown |  |
| 6 | M148T21 | 148.0604 | 130.0505 (51.7)  87.0442 (1.8)  84.0447 (5.5) | C_5_H_9_NO_4_ | Glutamate |  |
| 7 | M271T560 | 271.2744 | 71.0862 (100)  165.0141 (16.3) |  | Unknown |  |
| 8 | M297T48 | 297.0812 | 87.0443 (86.3)  105.0547 1792  175.0238 (100) |  | Unknown |  |
| 9 | M259T82 | 259.0786 | 125.0707 (100)  148.0626 (50) | C_13_H_12_N_3_OS_1_ | Unknown, reported in the literature (Steuer et al., 2019) | 3 |
| 10 | M119T79 | 119.0352 | 77.0152 (100)  94.0422 (46.3)  65.0166 (49.3) | C_4_H_6_O_4_ |  |  |
| 11 | M428T460 | 428.3737 | 85.0290 (100)  144.1011 (31.5)  369.3096 (11.0) | C_25_H_49_NO_4_ | Unknown, 4-Methylheptadecanoylcarnitine | 2 |
| 12 | M130T173 | 130.0498 | 84.0437 (100)  56.05 (12.2)  68.9984 (14.4) |  | Unknown |  |
| 13 | M468T432 | 468.3089 | 104.1081 (13.7)  184.0728 (100)  450.2975 (5.8) |  | Unknown |  |
| 14 | M560T479 | 560.3109 | 104.1060 (67.4)  501.2432 (62.9)  339.2883 (30.1) |  | Unknown |  |
| 15 | M456T478 | 456.4044 | 85.0312 (77.3)  131.0902 (100) | C_27_H_53_NO_4_ | Arachidylcarnitine | 2 |
| 16 | M534T471 | 534.2952 | 171.1008 (91.9)  444.9476 (98.9)  498.8389 (44.8) |  | Unknown |  |
| 17 | M146T128 | 146.0602 | 117.0571 (100)  91.0554 (26.1)  143.0730 (28.6)  65.0401 (10.6) | C_9_H_7_NO | Indole-3-carboxaldehyde  (HMDB0029737) fragment fit with database | 2 |
| 18 | M534T471 | 534.2952 | 104.1133 (100)  129.0695 (34.0) |  | unknown |  |
| 19 | M271T560 | 271.2744 | 132.9922 (67.6)  183.1181 (100) |  | Unknown |  |
| 20 | M494T442 | 494.3244 | 311.2594 (3.2)  184.0752 (100)  104.1090 (12.3)  86.0970 (4.29) |  | Unknown |  |
| 21 | M560T479 | 560.3109 | 104.1052 (100)  124.1200 (64.3)  184.0757 (54.5) |  | Unknown |  |
| 22 | M363T516 | 363.1721 | 153.0701 (65.7)  251.0497 (100) |  | Unknown |  |
| 23 | M234T52 | 234.0969 | 84.0455 (21.9)  130.0496 (100) | C_9_H_15_NO_6_ | GABA-2-hydroxyglutarate | 2 |
| 24 | M410T173 | 410.0775 | 184.0738 (100)  127.0352 (37.8) |  | Unknown |  |
| 25 | M118T128 | 118.0653 | 117.0585 (100)  91.0546 (18.2)  65.0390 (30.1) |  | Unknown |  |
| 26 | M118T24 | 118.0868 | 58.0666 (98.8)  70.0627 (2.1) | C_5_H_11_NO_2_ | Betaine | 1 |
| 27 | M116T22_2 | 116.0711 | 58.0680 (1.2)  70.0671 (100) | C_5_H_9_NO_2_ | Proline | 1 |
| 28 | M134T23 | 134.0271 | 58.9964 (100)  88.0217 (23.4) | C_4_H_7_NO_2_S | Thioproline | 1 |
| 29 | M508T489 | 508.3765 | 104.1087 (100)  184.0740 (29.9)  86.0967 (14.5) | C_26_H_54_NO_6_P | Octadecenyl-sn-glycero-3-phosphocholine | 1 |
| 30 | M298T130 | 298.0968 | 136.0634 (100)  119.0364 (7.6)  75.0272 (4.5) | C_11_H_15_N_5_O_3_S | MTA | 1 |
| 31 | M130T173 | 130.0498 | 57.9368 (100)  73.0687 (96.8)  81.0321 (74.7) |  | Unknown |  |
| 32 | M372T413 | 372.3110 | 85.0296 (100)  144.1029 (64.3)  211.2074 (75.4)  313.2413 (97.4) | C_21_H_41_NO_4_ | Myristorylcarnitine | 1 |
| 33 | M105T392 | 105.0702 | 65.0422 (100)  120.9773 (73.5) |  | Unknown |  |
| 34 | M534T471 | 534.2952 | 105.1136 (100)  183.0405 (96.6)  322.8764 (88.5) |  | Unknown |  |
| 35 | M426T443 | 426.3583 | 85.0295 (100)  60.0821 (10.7)  144.1032 (17.4) | C_25_H_47_NO_4_ | Oleoylcarnitine | 1 |
| 36 | M262T52 | 262.0134 | 84.0457 (25.1)  130.0512 (100)  137.0451 (29.3) |  | Unknown |  |
| 37 | M538T535 | 538.3872 | 184.0703 (100)  365.7641 (26.6)  146.1185 (23.4) |  | Unknown |  |
| 38 | M91T60 | 91.0541 | 62.0019 (100)  75.0226 (56.5) |  | Unknown |  |
| 39 | M200T23 | 200.1281 | 85.0283 (100)  182.1195 (12.4)  70.0666 (69.8) |  | Unknown |  |
| 40 | M482T481 | 482.3479 | 86.0972 (12.0)  104.1081 (65.5)  125.0006 (78.3)  184.0738 (100) |  | 1-O-Hexadecyl-lyso-sn-glycero-3-phosphocholine (KEGG) | 2 |
| 41 | M484T74 | 484.1164 | 137.0454 (100)  235.0232 (47.7)  153.0627 (59.9) |  | Unknown |  |
| 42 | M130T246 | 130.0651 | 69.0686 (23.4)  117.0701 (13.9) |  | Unknown |  |
| 43 | M572T482 | 572.3711 | 184.0721 (100)  421.1448 (20.2)  104.1092 (20.7) |  | Unknown |  |
| 44 | M510T491 | 510.3559 | 184.0721 (100)  105.0770 (45.8)  404.7829 (36.0) | C_25_H_52_NO_7_P | Lysophosphatidylcholine 17:0 | 1 |
| 45 | M132T128 | 132.0807 | 117.0583 (100)  77.0389 (13.3)  90.0448 (12.7) |  | Unknown |  |
| 46 | M560T479 | 560.3109 | 104.1045 (100)  276.8110 (73.3)  451.8436 (47.4) |  | Unknown |  |
| 47 | M138T112 | 138.0913 | 80.0513 (100)  94.0658 (96.7)  60.9944 (69.6) |  | Unknown |  |
| 48 | M316T356 | 316.2493 | 85.0296 (100)  144.1027 (29.8) | C_17_H_33_NO_4_ | Decanoylcarnitine | 1 |
| 49 | M199T92 | 199.1076 | 70.0660 (100)  107.0896 (77.0)  159.0775 (66.7) | C₉H₁₄N₂O₃ | Cyclo (Pro-Thr) | 1 |
| 50 | M147T80 | 147.1127 | 60.9889 (100)  84.0818 (62.8) | C_6_H_14_N_2_O_2_ | Lysine | 1 |
| 51 | M204T23 | 204.1238 | 85.0300 (100)  144.1009 (10.8) | C_9_H_17_NO_4_ | Acetylcarnitine | 1 |
| 52 | M153T52 | 153.0407 | 82.0396  110.0357 | C_5_H_4_N_4_O_2_ | Xanthine | 1 |
| 53 | M363T516 | 363.1721 | 251.0497 (100)  153.0701 (65.7)  215.0260 (26.9)  71.0855 (20.4) |  | Unknown |  |
| 54 | M385T74 | 385.1289 | 136.0632 (100)  90.0150 (15.8) | C_14_H_20_N_6_O_5_S | 5-adenosyl- homocysteine | 1 |
| 55 | M148T21 | 148.0604 | 84.0482 (100)  117.0659 (52.3) | C_5_H_9_NO_4_ | Glutamic acid |  |
| 56 | M262T41 | 262.1285 | 137.0469 (100)  123.0556 (41.3)  130.0519 (10.5)  85.0275 (2.8) |  | Unknown |  |
| 57 | M197T159 | 197.1285 | 70.0683 (100)  124.1126 (92.2) | C₁₀H₁₆N₂O₂ | Cyclo (Pro-Val) | 1 |
| 58 | M147T21 | 147.0764 | 84.0450 (100)  139.0230 (21.1)  56.0494 (44.7) | C_5_H_10_N_2_O_3_ | Glutamine | 1 |
| 59 | M220T109 | 220.1196 | 124.0781 (100)  69.0703 (59.9) | C_9_H_17_NO_5_ | Pantothenic acid | 1 |
| 60 | M288T319 | 288.2169 | 85.0302 (100)  144.1009 (14.0) | C_15_H_29_NO_4_ | Octanoylcarnitine | 1 |
| 61 | M522T480 | 522.3569 | 104.1082 (8.4)  184.0752 (100)  258.1107 (3.1) | C_26_H_52_NO_7_P | L-a-Lysophosphatidylcholine (L-A-LysoPC); 18:1 | 1 |
| 62 | M265T151 | 265.1545 | 120.0819 (100)  72.0787 (7.3) | C_14_H_20_N_2_O_3_ | Phe-Val | 1 |
| 63 | M344T387 | 344.2798 | 85.0298 (100)  144.1032 (72.7) | C_19_H_37_NO_4_ | Launoylcarnitine | 1 |
| 64 | M176T283 | 176.0706 | 130.0650 (100)  103.0554 (5.6) | C_10_H_9_NO_2_ | 3-indole-acetic-acid | 1 |
| 65 | M206T246 | 206.0810 | 118.0663 (100)  130.0654 (90.0)  170.0558 (13.0) | C_11_H_11_NO_3_ | 3-Indole lactic acid | 1 |
| 66 | M144T130 | 144.1015 | 117.0530 (88.1)  91.0546 (76.2)  59.0496 (84.2) |  | Unknown |  |
| 67 | M231T428 | 231.01743 | 72.0806  118.0865  132.1018 | C_11_H_22_N_2_O_3_ | Val-Leu | 2 |
| 68 | M118T128 | 118.0653 | 65.0390 (30.1)  91.0546 (18.2)  117.0585 (100) |  | Unknown |  |

Note: samples were fragmented in either DDA or DIA mode. As not all the selected precursors got fragments in auto-ms/ms mode, fragments without abundancy in table mean that they were fragmented in bbcid mode, otherwise they were fragmented in auto-MS/MS mode.

**Figures**


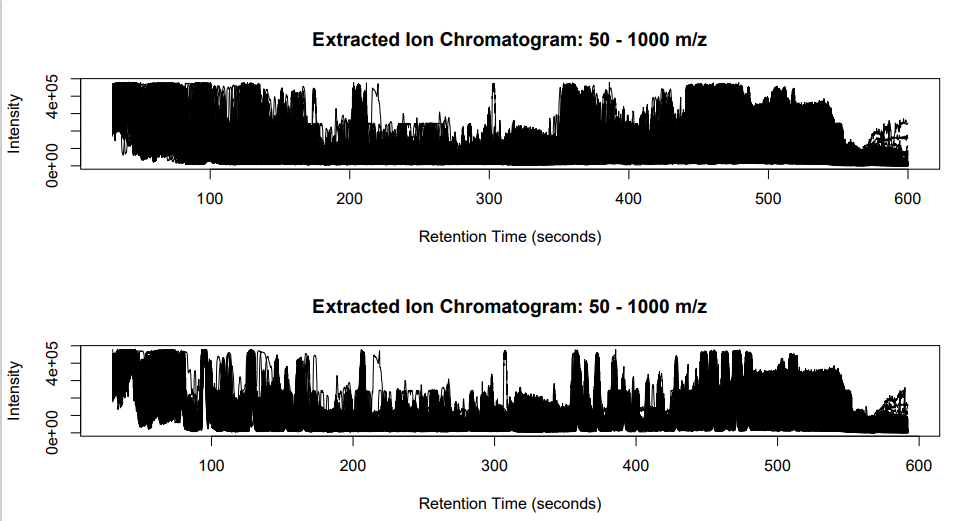


After alignment

Before alignment

**Figure** S1. Retention correction of all studied samples (before and after peak alignment)


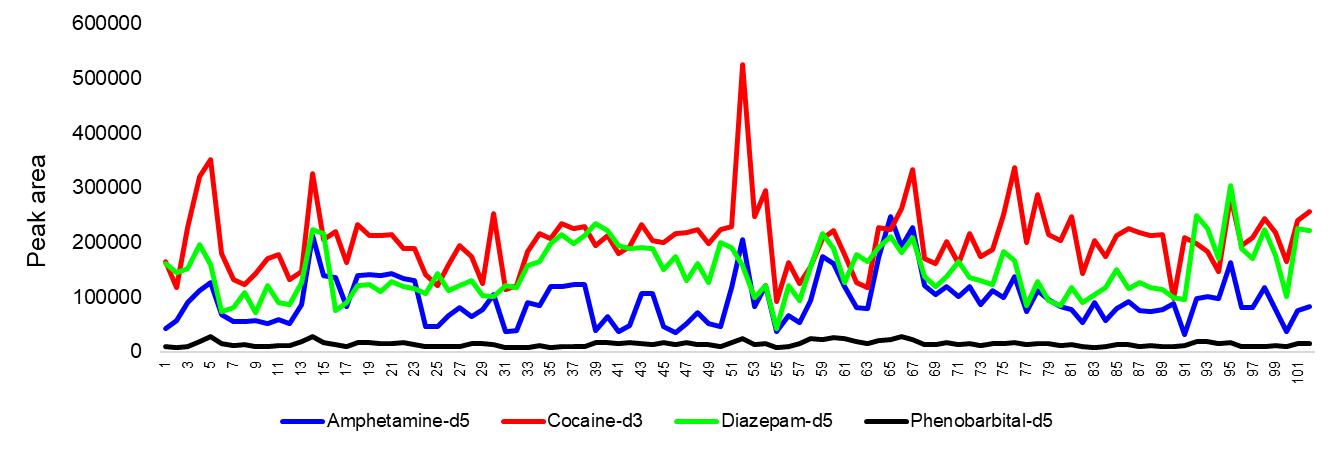


**Figure** S2. Variation of internal standards in all the studied samples. It shows variation before NOMIS normalization, and these four internal standards were not in the output after NOMIS. Four internal standards were applied in all the collected samples along the six years, and the variation could be up to five times.

**Figure** S3. Accuracy of GHB peak area integrated by XCMS. X axis is the manually integrated peak area of GHB of positive samples, the y axis is the xcms integrated peak area of GHB of positive samples.


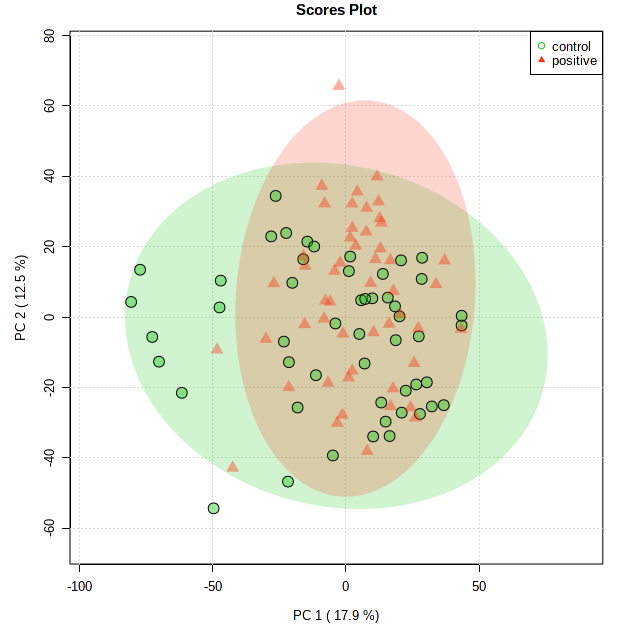


**Figure** S4. PCA plot of GHB positive and control samples


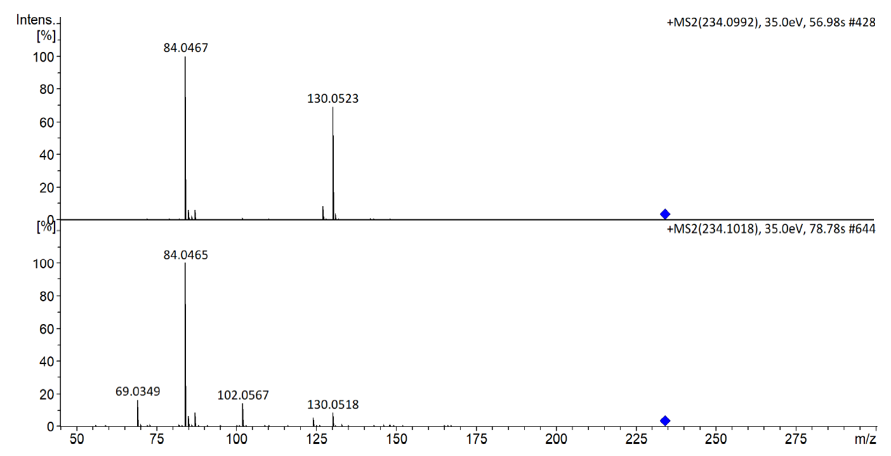

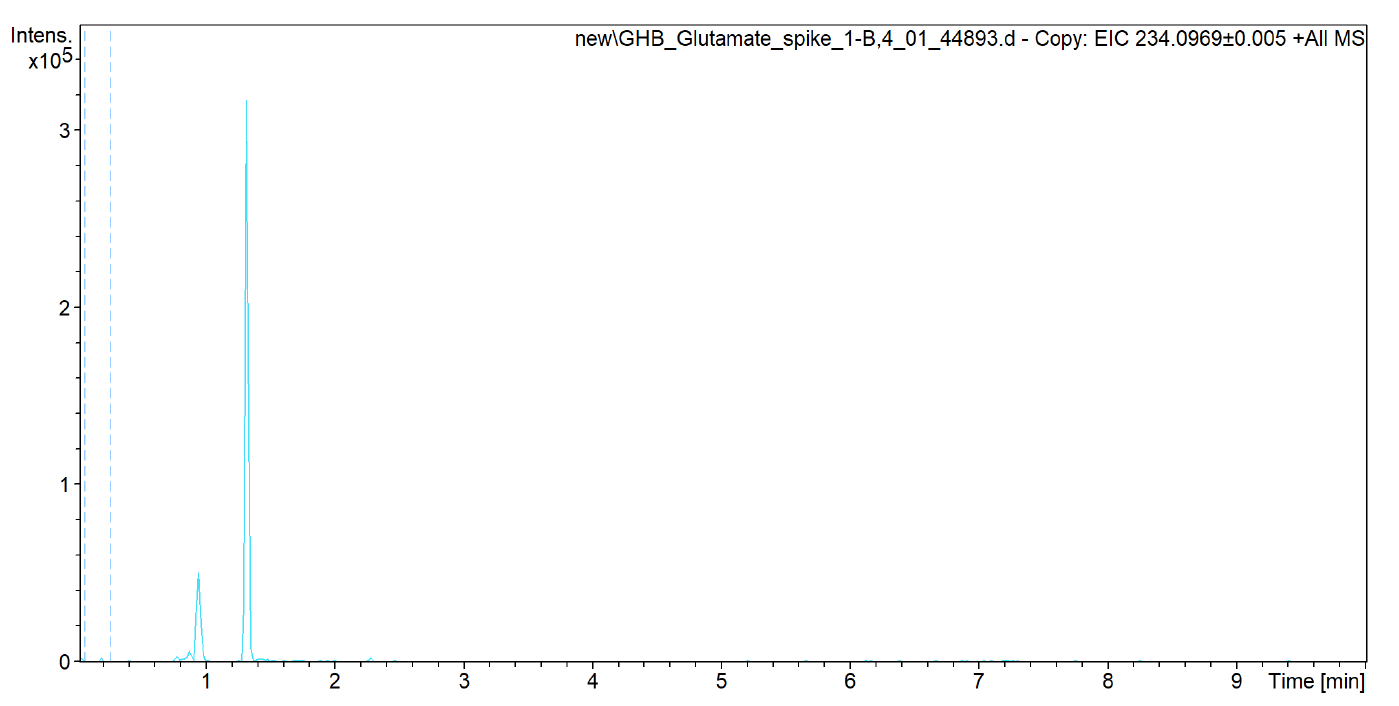


Synthesized GHB glutamate

Feature

M234T52

**Figures** S5. Spectrum of GHB positive sample spiked with GHB-glutamate that obtained by auto MS/MS mode. The delta RT of these two compounds with same mass is about 0.4 min.

**Others**

**Synthesis of GHB glutamate**

***Method and materials***

Chemicals and solvents were obtained from Sigma-Aldrich, TCI, Fluorochem or Merck, and were used as received. Thin layer chromatography (TLC) was performed on silica gel pre-coated aluminum-backed sheets (0.2 mm, Merck silica gel 60 F_254_). Visualization of the TLC sheets was accomplished by UV light at 254 nm and/or KMnO_4_ stain. Column chromatography was performed using high-purity grade silica gel (60 Å, 230-400 mesh) and high-purity grade solvents. NMR spectra were acquired on a Varian AS400 spectrometer running at 400 MHz for ^1^H and 100 MHz for ^13^C. Chemical shifts (δ) are reported in ppm relative to residual solvent signals (^1^H NMR: CHCl_3_ @ 7.26 ppm; DMSO-*d_6_* @ 2.50 ppm. ^13^C NMR: CDCl_3_ @ 77.16 ppm; DMSO-*d_6_* @ 39.52 ppm). The following abbreviations are used to indicate the multiplicity in the NMR spectra: s, singlet; d, doublet; t, triplet; q, quartet; m, multiplet; bs, broad signal. ^13^C NMR spectra were acquired in broad band decoupled mode. Electrospray ionization high-resolution mass spectrometry (ESI-HRMS) were performed a Q-TOF maXis Impact mass spectrometer (Bruker Daltonik GmbH, Germany) operated in positive (4.0 kV) or negative (2.5 kV) mode.


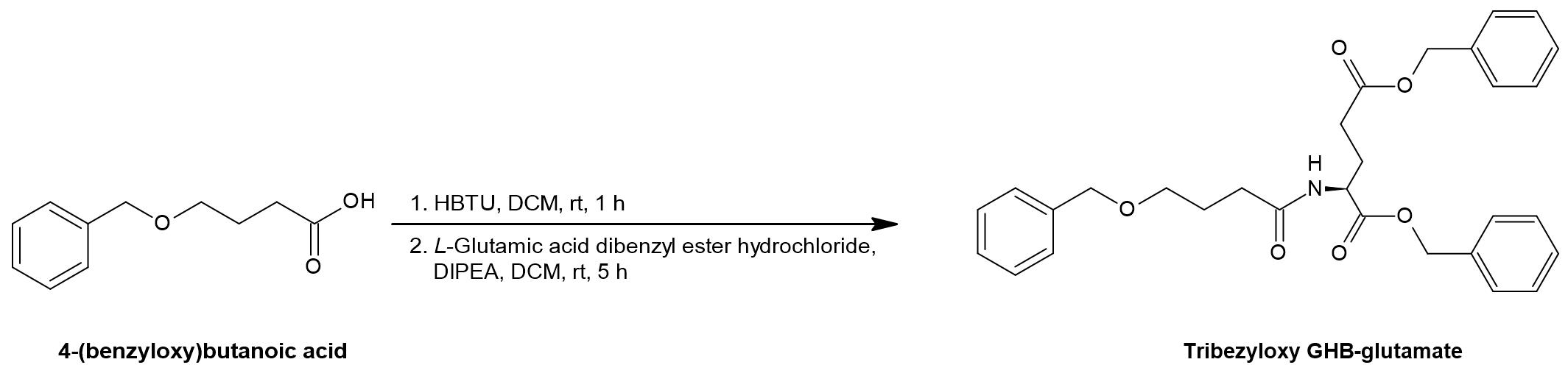


**Figure** S6. Synthesis of tribenzyloxy GHB-glutamate

4-(benzyloxy)butanoic acid (250 µL; 1.41 mmol) was dissolved in anhydrous dichloromethane (DCM, 5 mL), hexafluorophosphate benzotriazole tetramethyl uronium (HBTU; 535 mg; 1.41 mmol) was added, and the resulting suspension was stirred at room temperature for 1 hour. A solution of *L*-glutamic acid dibenzyl ester hydrochloride (514 mg; 1.41 mmol) and diisopropylethylamine (DIPEA; 492 µL; 2.82 mmol) in anhydrous DCM (2 mL) was added and the reaction mixture was stirred at room temperature for 5 hours until complete by TLC (50% EtOAc in hexane; R_f_ = 0.28). The reaction mixture was concentrated *in vacuo* and the product was purified by silica chromatography using 50% EtOAc in hexane as eluent. The product tribenzyloxy GHB-glutamate was obtained as a clear oil (700 mg; 99% yield).

^1^H NMR (400 MHz, CDCl_3_) δ (ppm) 7.34 – 7.18 (m, 15H), 6.27 (d, *J* = 7.8 Hz, 1H), 5.10 (s, 2H), 5.03 (s, 2H), 4.60 (td, *J* = 7.9, 5.0 Hz, 1H), 4.43 (s, 2H), 3.45 (t, *J* = 6.0 Hz, 2H), 2.42 – 2.28 (m, 2H), 2.27 (t, *J* = 7.6 Hz, 2H), 2.20 – 2.09 (m, 1H), 1.96 – 1.85 (m, 1H), 1.87 (quintet, *J* = 6.4 Hz, 2H).

^13^C NMR (100 MHz, CDCl_3_) δ (ppm) 172.9, 172.7, 171.9, 138.5, 135.9, 135.3, 128.8, 128.7, 128.6, 128.54, 128.45, 128.43, 128.4, 127.9, 127.8, 73.1, 69.4, 67.4, 66.7, 51.8, 33.4, 30.4, 27.4, 25.6.

ESI-HRMS: obs. *m/z* 504.2396 (504.2381 calcd. for [M + H]^+^).


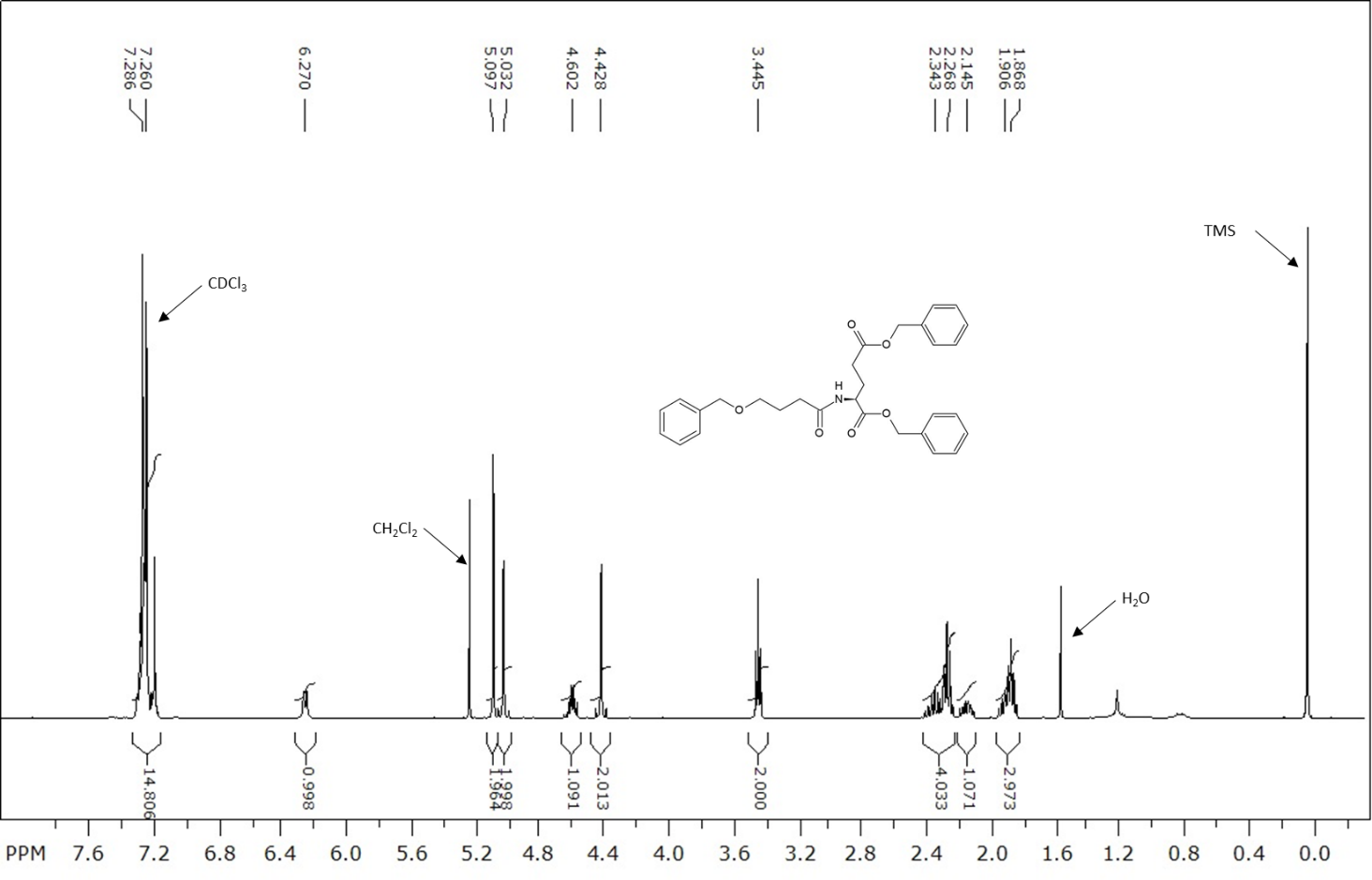


**Figure** S7. ^1^H NMR (400 MHz, CDCl_3_) of tribenzyloxy GHB-glutamate.


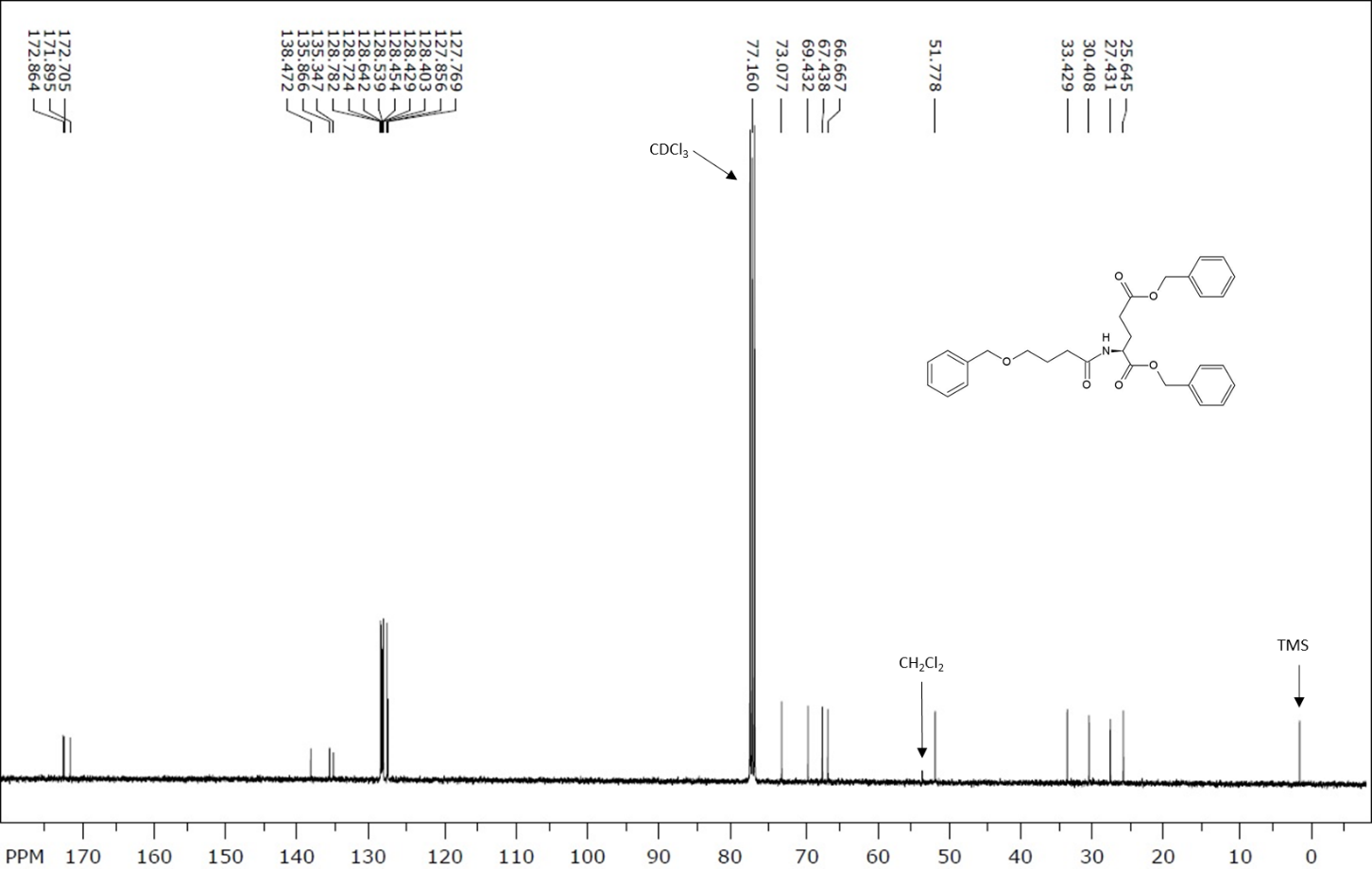


**Figure** S8. ^13^C NMR (100 MHz, CDCl_3_) of tribenzyloxy GHB-glutamate

**Synthesis of GHB-glutamate**

**Figure** S9. Synthesis of GHB-glutamate

Tribenzyloxy GHB-glutamate (103 mg; 0.20 mmol) was dissolved in methanol (5 mL) and 10 wt% Pd/C (21 mg) was added. The reaction flask was repeatedly evacuated and filled with hydrogen gas 6 times, then left with a ballon of H_2_ gas stirring at room temperature for 21 h until complete by TLC (50% EtOAc in hexane; R_f_ = 0). The reaction mixture was filtered through Celite by suction (washing with 2 x 10 mL methanol), then filtered by gravity and evaporated *in vacuo* to give the product GHB-glutamate as a clear oil (47 mg; 99% yield).

^1^H NMR (400 MHz, DMSO-*d*_6_) δ (ppm) 8.00 (d, *J* = 7.8 Hz, 1H), 4.22 – 4.12 (m, 1H), 3.37 (t, *J* = 6.5 Hz, 2H), 2.25 (t, *J* = 7.6 Hz, 2H), 2.14 (t, *J* = 6.9 Hz, 2H), 1.98 – 1.86 (m, 1H), 1.82 – 1.69 (m, 1H), 1.62 (quintet, *J* = 6.9 Hz, 2H).

^13^C NMR (100 MHz, DMSO-*d*_6_) δ (ppm) 173.8, 173.5, 172.3, 60.3, 51.2, 31.8, 30.3, 28.6, 26.5.

ESI-HRMS: obs. m/z 234.0957 (234.0972 calcd. for [M + H]^+^).

ESI-HRMS: obs. *m/z* 232.0834 (232.0827 calcd. for [M - H]^-^).


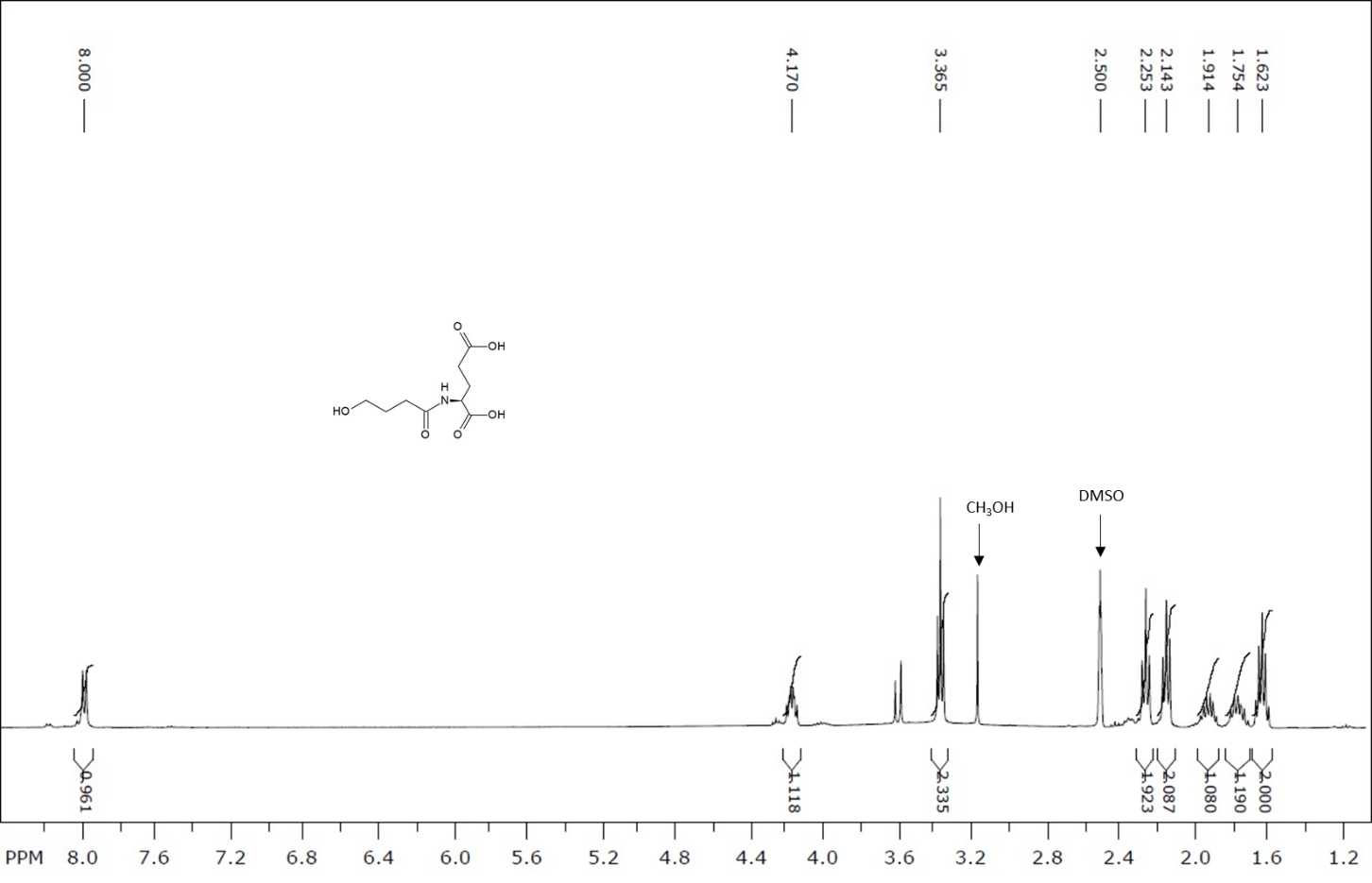


**Figure** S10. ^1^H NMR (400 MHz, DMSO-*d_6_*) of GHB-glutamate


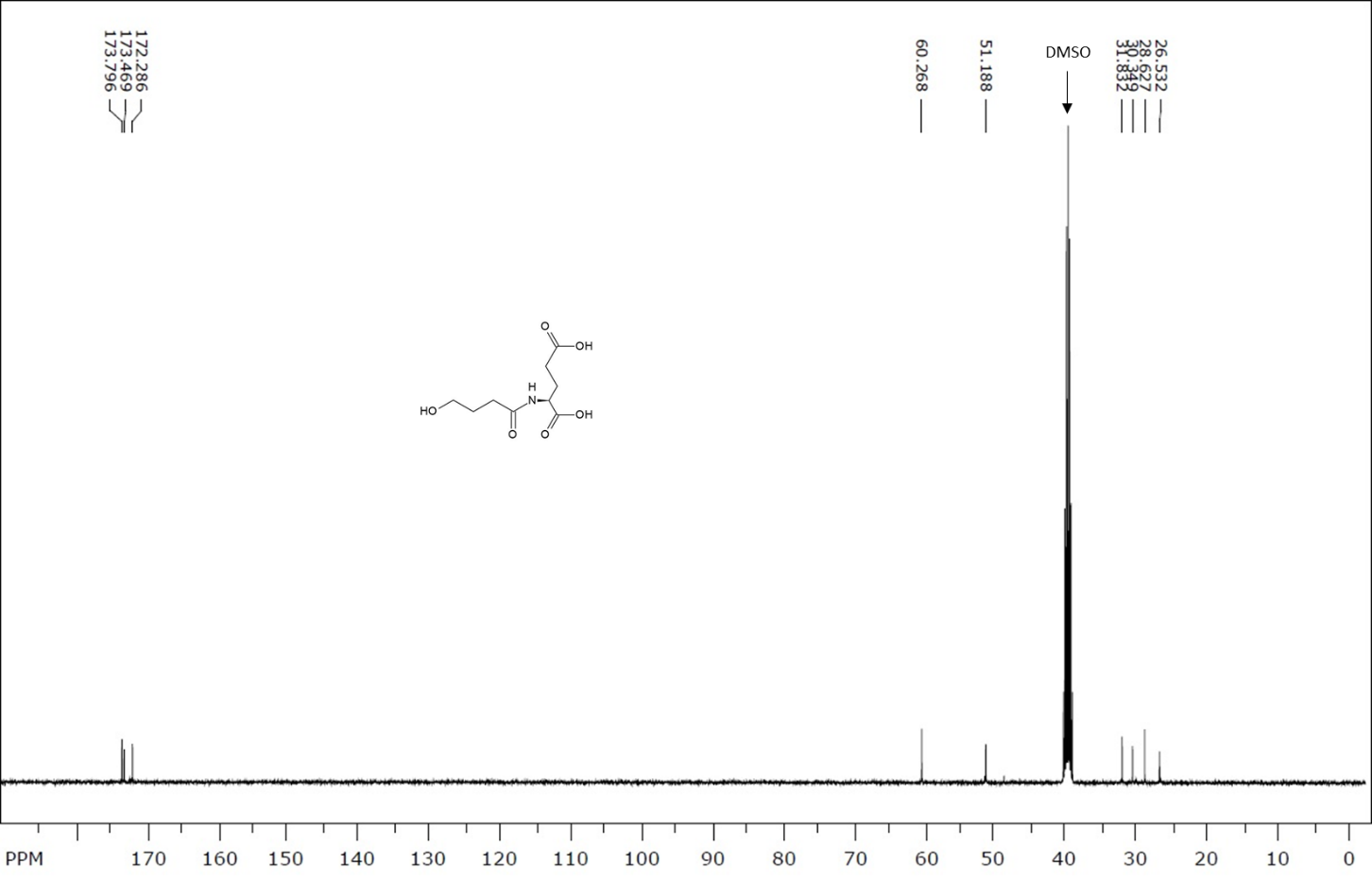


**Figure** S11. ^13^C NMR (100 MHz, DMSO-*d*_6_) of GHB-glutamate


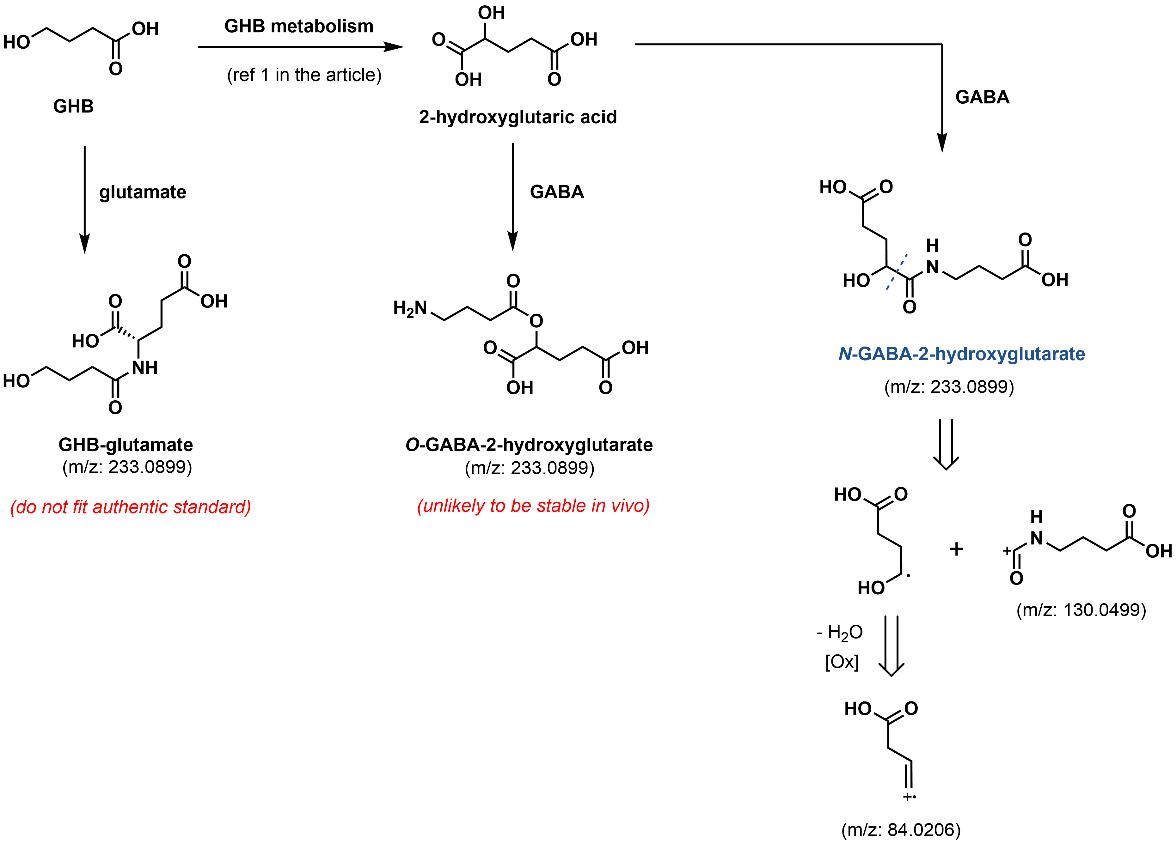


**Figure** S12. Proposed structure of M234T52 and possible pathway
